# Mitochondrial Dysfunction in Apoptosis-Resistant Acute Myeloid Leukemia Cells During a Sterile Inflammatory Response

**DOI:** 10.3390/biom15121635

**Published:** 2025-11-21

**Authors:** Elena I. Meshcheriakova, Kirill S. Krasnov, Irina V. Odinokova, Aleksey I. Lomovsky, Olga V. Krestinina, Yuliya L. Baburina, Irina B. Mikheeva, Gulnara Z. Mikhailova, Anatoly S. Senotov, Polina S. Nekhochina, Yana V. Lomovskaya, Vladislav V. Minaychev, Irina S. Fadeeva, Margarita I. Kobyakova, Roman S. Fadeev

**Affiliations:** 1 Institute of Theoretical and Experimental Biophysics, Russian Academy of Sciences, 142290 Pushchino, Russia; elena.mesh2311@gmail.com (E.I.M.); kirill.krasnov64@gmail.com (K.S.K.); odinokova@rambler.ru (I.V.O.); lomovskyalex@gmail.com (A.I.L.); ovkres@mail.ru (O.V.K.); byul@rambler.ru (Y.L.B.); mikheirina@yandex.ru (I.B.M.); gulnaramari@yandex.ru (G.Z.M.); a.s.senotov@gmail.com (A.S.S.); yannalomovskaya@gmail.com (Y.V.L.); vminaychev@gmail.com (V.V.M.); aurin.fad@gmail.com (I.S.F.); fadeevrs@gmail.com (R.S.F.); 2 Institute of Cell Biophysics of the Russian Academy of Sciences, Federal Research Center “Pushchino Scientific Center for Biological Research of the Russian Academy of Sciences”, 142290 Pushchino, Russia; 3 Research Institute of Clinical and Experimental Lymphology—Branch of Institute of Cytology and Genetics, Siberian Branch of Russian Academy of Sciences, 630060 Novosibirsk, Russia; 4 Faculty of Biotechnology, Lomonosov Moscow State University, 119234 Moscow, Russia; polinanekh@yandex.ru

**Keywords:** mitochondrial dysfunction, proinflammatory activation, acute myeloid leukemia, drug resistance

## Abstract

Mitochondria are crucial for energy metabolism and the regulation of apoptosis and the inflammatory response in acute myeloid leukemia (AML). This study examined key mitochondrial characteristics in apoptosis-resistant AML cells during in vitro aseptic pro-inflammatory activation utilizing spectrofluorimetry, quantitative reverse transcription PCR, Western blotting, differential gene expression analysis, flow cytometry, transmission electron microscopy, and cellular respiration analysis. Under conditions of aseptic inflammation simulated in three-dimensional high-density cultures, apoptosis-resistant AML cells exhibited a significant reduction in the transcriptional activity of genes linked to oxidative phosphorylation and the tricarboxylic acid cycle; demonstrated diminished mitochondrial respiration activity; and decreased levels of the mitophagy regulatory proteins PINK1 and Parkin. Furthermore, pathogenic alterations in mitochondrial morphology were observed. These cells demonstrated enhanced intracellular generation of reactive oxygen species, lactate accumulation in the culture media, elevated levels of DRP1 protein, and an increased fraction of small and medium-sized mitochondria. The acquired data demonstrate that aseptic pro-inflammatory activation results in metabolic remodelling of acute myeloid leukemia cells, integrating characteristics of mitochondrial dysfunction. This condition may facilitate the persistence of leukemic cells during inflammatory stress and potentially contribute to the development of an apoptosis-resistant phenotype. The established in vitro model is crucial for examining both the characteristics of energy metabolism and the anti-apoptotic mechanisms in leukemic cells.

## 1. Introduction

Acute myeloid leukemia (AML) is a severe hematological malignancy originating from myeloid stem and progenitor cells, leading to the buildup of immature myeloid blasts in the bone marrow [1]. Acute myeloid leukemia (AML) is the predominant form of acute leukemia, representing over 80% of cases [2]. Chemotherapy is presently the primary treatment for this condition [3]. Nonetheless, the emergence of drug resistance in AML cells constrains the efficacy of this strategy.

Mitochondria are cellular organelles that govern essential tasks, such as ATP and NADH generation, metabolic processes, intracellular signalling, and homeostasis maintenance in both normal and altered cells [4,5]. Numerous investigations have established that mitochondria play a crucial role in the initiation, development, and metastasis of malignant neoplasms [4,5,6]. Moreover, they play a pivotal role in the emergence of drug resistance in cancer cells by modulating apoptosis via cytochrome C-dependent apoptosome formation [7,8,9].

A burgeoning corpus of research indicates that mitochondria play a crucial role in the regulation and initiation of inflammatory reactions [9,10,11]. Chronic aseptic inflammation, a characteristic of cancer [1,12], is linked not only to solid tumours but also to the advancement of myelodysplastic syndrome to acute myeloid leukemia (AML) [1,13]. Tumour-associated inflammation influences practically all phases of carcinogenesis, encompassing the origin and proliferation of the main tumour, as well as metastasis, therapeutic resistance, and recurrence [14,15]. Inflammatory mediators generated by immunological and malignant cells can facilitate cancer progression and resistance to therapy [14,16] by activating survival signalling pathways, such as JAK/STAT or NF-κB [14,17]. Mitochondrial protein-nucleic acid complexes demonstrate considerable structural resemblance to bacterial molecules, enabling them to function as potential ligands for pattern recognition receptors (DAMPs) [9,10,18]. Mitochondrial malfunction or injury can consequently induce aseptic inflammatory responses. This transpires through the activation of the cGAS/STING signalling pathway by mitochondrial DNA and by inflammasome activation prompted by mtDNA and reactive oxygen species (ROS) [9,10].

Previously, we developed and described an in vitro model of a sterile inflammatory response in AML cells using high-density three-dimensional cultures. In this model, AML cells were shown to produce pro-inflammatory cytokines and chemokines and to express surface CD markers characteristic of pro-inflammatory activation [19]. Furthermore, AML cells in these high-density three-dimensional cultures demonstrated sustained resistance to apoptosis [19]. It is well known that the bone marrow in AML is a damaged tissue with signs of chronic inflammation. This inflammatory microenvironment can help tumor cells avoid cell death and is also a marker of poor prognosis [20,21]. The present study examines the mitochondrial status of apoptosis-resistant AML cells under conditions of sterile pro-inflammatory activation using this model.

## 2. Materials and Methods

### 2.1. Cell Culture and Conditions

Human AML THP-1, MV4-11, HL-60 cells were obtained from the American Type Culture Collection (ATCC, Manassas, VA, USA). Cells were cultured in RPMI-1640 medium (Sigma-Aldrich, St. Louis, MO, USA) supplemented with 10% fetal bovine serum (FBS; Gibco, Thermo Fisher Scientific, Waltham, MA, USA) and 40 μg/mL gentamicin sulfate (Sigma-Aldrich, St. Louis, MO, USA). Cultures were maintained at 37 °C in a humidified atmosphere with 5% CO_2_. To obtain low-density cultures (LDC) of apoptosis-sensitive cells, 5 × 10^3^ cells were seeded in 100 μL of culture medium per well of a U-bottom 96-well plate (Corning Inc., Corning, NY, USA). After 24 h, the cell count increased to 1.0 ± 0.1 × 10^4^ cells per well (equivalent to 1.0 ± 0.1 × 10^5^ cells/mL). To generate apoptosis-resistant, three-dimensional high-density cultures simulating aseptic proinflammatory cell activation (HDC), 5 × 10^3^ cells were seeded in 100 μL of medium per well in a U-bottom 96-well plate and cultured for 5 days without medium change. At the end of the culture period, each well contained a single three-dimensional multicellular aggregate with a cell count of 1.0 ± 0.1 × 10^5^ cells (equivalent to 1 × 10^6^ cells/mL) [19,22,23]. Cells stimulated with lipopolysaccharide (LPS), a known proinflammatory activator, were also included in the study [24,25,26]. For this purpose, cells were seeded at a density of 5 × 10^4^ cells/mL in a culture medium containing 10 µg/mL of LPS from *E. coli* O111:B4 (Santa Cruz Biotechnology, Dallas, TX, USA) and incubated for 24 h. After 24 h, the cell density increased to 9.0 ± 0.5 × 10^4^ cells/mL. Cells with depleted mtDNA (ρ^0^ cells) were generated by culturing them with 10 μM dideoxycytidine (MACKLIN, Shanghai, China) and 100 μg/mL uridine (Pallav Chemicals, Mumbai, India) for 7 days [27,28]. For this purpose, 2.5 × 10^5^ cells were seeded in 5 mL of culture medium. After 24 h, dideoxycytidine and uridine were added to the medium. The cells were incubated for 7 days, replacing the medium every 48 h with a fresh one with the same concentrations of dideoxycytidine and uridine. After incubation, the number of cells was 8.0 ± 0.6 × 10^5^ cells/mL. The proportion of dead cells in each group, as determined by staining with 0.2% trypan blue (Sigma-Aldrich, St. Louis, MO, USA), did not exceed 5%.

### 2.2. Cell Viability Assay

To produce high-density, apoptosis-resistant three-dimensional cultures (HDC) that replicate the sterile inflammatory activation of proinflammatory cells, 5 × 10^3^ cells in 100 µL of media were inoculated onto U-bottom 96-well plates and incubated for 5 days without medium replenishment. At the conclusion of the culture period, each well housed a solitary three-dimensional multicellular aggregate with a final cell count of (1.0 ± 0.1) × 10^5^ cells per well, corresponding to a density of 1 × 10^6^ cells/mL. Thereafter, the cells were pre-incubated with 1 µM Navitoclax (Cell Signaling Technology, Inc., Danvers, MA, USA) for 4 h prior to the introduction of cell death inducers. Cell viability was determined by calculating the ratio of live cells in experimental cultures to those in control cultures (without drugs) after a 24-h incubation with etoposide (Selleckchem, Houston, TX, USA) or recombinant TRAIL protein, which was obtained as described previously [29,30]. The number of viable cells following drug treatment was quantified by measuring the intensity of resazurin reduction (Sigma-Aldrich, St. Louis, MO, USA) using an Infinite F200 plate reader (Tecan, Männedorf, Switzerland) [19].

### 2.3. RNA Sequencing (RNA-Seq)

Total RNA sequencing was performed by “Genoanalitika” LLC using the Illumina HiSeq 1500 platform (San Diego, CA, USA). Sequencing was conducted for two experimental groups, each with three biological replicates. The raw and processed data are available in the Gene Expression Omnibus (GEO) database under accession number GSE298747 (https://www.ncbi.nlm.nih.gov/geo/query/acc.cgi?acc=GSE298747, accessed on 9 June 2025).

### 2.4. Differential Gene Expression Analysis

Differential gene expression analysis was performed using the DESeq2 package (version 1.38.3) in R (version 4.2.2) [31]. We compared the expression profiles of apoptosis-resistant THP-1 AML cells grown in high-density three-dimensional culture to those of apoptosis-sensitive cells grown in low-density culture. We used the design formula~condition, where “condition” represents the “HDC” and “LDC” samples, with no additional covariates included. The comparison was specified as “HDC” versus “LDC”. Using the DESeq2-normalized count matrix, we employed both a distance-based approach and Principal Component Analysis (PCA) to compare samples based on gene expression data by scipy (v. 1.11.4) and scikit-learn (v. 1.3.2) python packages respectively. For the distance-based analysis, pairwise Euclidean distances between samples were computed, followed by hierarchical clustering to generate similarity heatmaps. For the PCA, all differentially expressed genes were used to evaluate sample relationships through multivariate decomposition of variance.

### 2.5. Gene Set Enrichment Analysis (GSEA)

GSEA was performed using MitoPathways gene sets obtained from the MitoCarta3.0 database [32] using the GSEApy package (version 1.1.1) for the Python programming language (version 3.11) [33].

### 2.6. Mitochondrial Metabolism-Related Genes (MMRG) Expression and Functional Analysis

An annotated gene list from the MitoCarta3.0 database was used to identify MMRG [32]. From the genes that showed statistically significant differential expression (FDR < 0.05, |log2FoldChange| > 0) under high-density conditions, we selected those present in MitoCarta3.0. Functional enrichment analysis of the identified MMRG was performed with the MitoPathways gene sets (from the MitoCarta 3.0 database) via the Enrichr web service algorithms, which are integrated into the GSEApy package (version 1.1.1).

### 2.7. Construction and Analysis of a Protein–Protein Interaction (PPI) Network

A protein–protein interaction network for the products of the identified genes was constructed using stable Ensembl Gene IDs with the STRING database (version 12.0) [34]. The network was visualized using Cytoscape software (version 3.10.3) [35]. The analysis was restricted to genes whose products form a single connected network, thereby excluding isolated nodes. Network clustering was performed using the cytoHubba plugin (version 0.1) for Cytoscape, employing a combination of global and local node ranking algorithms to identify key hub genes [36].

### 2.8. Transmission Electron Microscopy (TEM)

For electron microscopy, a cell suspension was fixed for 3 h in a solution of 2.5% glutaraldehyde (NeoFroxx, Einhausen, Germany) in 0.1 M phosphate buffer (pH 7.2) at 4 °C. After fixation, the cells were washed three times with chilled 0.1 M phosphate buffer (pH 7.2) for 5 min each. Between washes, the cells were pelleted using a microcentrifuge (10,000 rpm, 1 min). The cell pellet was then post-fixed in 2% osmium tetroxide (OsO_4_) solution (Acros Organics, Geel, Belgium) for 1 h at 4 °C. Subsequently, the samples were dehydrated through a graded series of ethanol (30%, 50%, 70%, 96%, 100%) and pure acetone (100%) (Merck, Darmstadt, Germany), spending 10–15 min in each solution. The dehydrated samples were infiltrated and embedded in epoxy resin (Epon 812) (Clearsynth, Mumbai, India) according to a standard protocol, followed by polymerization at 37 °C for 24 h and then at 60 °C for another 24 h. Sections with a thickness of 60–70 nm were prepared from the polymerized blocks using a Leica EM UC6 ultramicrotome (Leica Microsystems, Wetzlar, Germany). The sections were sequentially stained with a 2% uranyl acetate solution (15–20 min, in the dark) (Pallav Chemicals, India) and with a lead citrate solution (Reynolds method, 5–10 min) (Honeywell International Inc., USA). The samples were examined using a JEM-1400+ transmission electron microscope (JEOL, Tokyo, Japan) operated at an accelerating voltage of 80 kV. Images were acquired at magnifications of ×3000 and ×10,000 and saved in TIFF format (8-bit, resolution 7904 × 6632 pixels). The micrographs were processed and morphometrically analyzed using the Image Tool 3.0 software (Informer Technologies, Inc., Los Angeles, CA, USA). The total number and size distribution of mitochondria, categorized as large (0.50–0.90 µm^2^), medium (0.19–0.28 µm^2^), and small (0.04–0.10 µm^2^), as well as the number of pathological forms, were quantified. Pathological features included reduced matrix electron density, complete or partial matrix vacuolization, disruption of mitochondrial membranes, and disorganization of cristae. The results are expressed as the count per plate (area of 10 µm^2^). The total number of mitochondria, along with the numbers of large (0.50–0.90 µm^2^), medium (0.19–0.49 µm^2^), small (0.04–0.18 µm^2^) mitochondria, and their pathological forms—characterized by partially or completely destroyed cristae—were counted and expressed as the number per plate area (per 10 µm^2^). For each experimental group, at least 100 micrographs were analyzed. Morphometric data were analyzed using Prism software for Windows (version 5.0). Cells with depleted THP1-ρ^0^ mtDNA were used as a positive control.

### 2.9. Mitochondrial Mass Analysis

To assess the mitochondrial mass, cells were stained with 5 µM Nonyl Acridine Orange (NAO) (Fisher Scientific, Waltham, MA, USA) for 30 min at 37 °C in an atmosphere of 5% CO_2_. The analysis was performed by flow cytometry using a BD Accuri C6 flow cytometer (BD Biosciences, Franklin Lakes, NJ, USA) [37]. Samples containing the same number of cells in each experimental group were prepared for analysis. Cells with depleted THP1-ρ^0^ mtDNA were used as a positive control.

### 2.10. Determination of Mitochondrial Membrane Potential (ΔΨm)

To determine the mitochondrial membrane potential (ΔΨm), cells were stained with 10 nM DiOC6(3) (Sigma-Aldrich, St. Louis, MO, USA) for 30 min at 37 °C in a 5% CO_2_ atmosphere. As a positive control, cells pre-incubated with 5 µM FCCP (Sigma-Aldrich, St. Louis, MO, USA) for 30 min were used. Analysis was performed by flow cytometry using a BD Accuri C6 flow cytometer [38,39]. Samples containing the same number of cells in each experimental group were prepared for analysis.

### 2.11. Determination of Intracellular Reactive Oxygen Species (ROS) Production

To determine intracellular reactive oxygen species (ROS) production, cells were stained with 20 µM 2′,7′-dichlorodihydrofluorescein diacetate (DCFH-DA) (Sigma-Aldrich, St. Louis, MO, USA) for 15 min in a CO_2_ incubator. As a positive control, cells pre-incubated with 1 mM hydrogen peroxide (Sigma-Aldrich, St. Louis, MO, USA) for 20 min were used. Fluorescence was measured using a BD Accuri C6 flow cytometer [39]. Samples containing the same number of cells in each experimental group were prepared for analysis.

### 2.12. Determination of Lactic Acid (Lactate) Concentration

To determine the lactic acid concentration in the culture medium, cells were centrifuged (300× *g*, 5 min), and the supernatant was collected for analysis. The measurement was performed using a commercial Lactic Acid Olvex diagnostic kit (Olvex Diagnostics, Moscow, Russia), according to the manufacturer’s instructions. The optical density was measured at a wavelength of 490 nm using an iMark microplate reader (Bio-Rad, Hercules, CA, USA). The obtained values were normalized to 1 × 10^5^ cells.

### 2.13. Evaluation of ADP/ATP and NAD/NADH Ratios

The ADP/ATP and NAD/NADH ratios were evaluated using the commercial ADP/ATP Ratio Assay Kit (Sigma-Aldrich, St. Louis, MO, USA) and the NAD/NADH Quantification Kit (Sigma-Aldrich, St. Louis, MO, USA), respectively, according to the manufacturer’s instructions. Measurements were performed using an Infinite F200 microplate spectrofluorimeter and an iMark microplate reader (Bio-Rad, Hercules, CA, USA), respectively. The obtained values were normalized to 1 × 10^5^ cells.

### 2.14. Analysis of Mitochondrial Respiration in Permeabilized Cells

Mitochondrial respiration was measured using an in situ model based on selective permeabilization of the plasma membrane with the nonionic detergent digitonin. Before the experiments, the optimal concentration of digitonin was carefully determined for each cell line to ensure effective plasma membrane permeabilization while preserving the integrity of the outer mitochondrial membrane. Integrity was assessed by monitoring cytochrome c release and the preservation of mitochondrial calcium uptake capacity. Measurements were performed in a thermostatically controlled 1 mL chamber equipped with a Clark oxygen electrode and Ca^2+^-selective electrode. The cell incubation medium contained the following components: 110 mM KCl, 5 mM NaCl, 10 mM HEPES (pH 7.4), and 5 mM KH_2_PO_4_ (all from Sigma-Aldrich, St. Louis, MO, USA). The cells (4 × 10^6^) were pelleted by centrifugation at 300× *g* for 3 min, resuspended in 1 mL of incubation medium, and transferred to a measurement chamber. The cells were then permeabilized by adding digitonin (0.05 mg/mL; 0.005%) (Sigma-Aldrich, St. Louis, MO, USA) to selectively permeabilize the plasma membrane without damaging the mitochondria. Mitochondrial respiration was supported by adding 10 mM succinate (Sigma-Aldrich, St. Louis, MO, USA) in the presence of 2 µM rotenone (Sigma-Aldrich, St. Louis, MO, USA). Based on the oxygen electrode readings, the following mitochondrial parameters were calculated: the basal respiration rate after adding digitonin to the cell suspension (State 2), the ADP-stimulated respiration rate (State 3) after adding 2 mM ADP (Sigma-Aldrich, St. Louis, MO, USA), and the uncoupled respiration rate after adding 30 µM DNP (2,4-dinitrophenol) (Sigma-Aldrich, St. Louis, MO, USA) [38].

### 2.15. Western Blot Analysis

Cell lysates were obtained using RIPA buffer (Santa Cruz Biotechnology, Dallas, TX, USA). Electrophoretic separation of proteins was performed in 12% polyacrylamide gels using a Mini-Protean Hoefer electrophoresis chamber (Pharmacia Biotech, Uppsala, Sweden) and a PowerPac Universal Power Supply (Bio-Rad, Hercules, CA, USA). Proteins were transferred to 0.2 µm nitrocellulose membranes (Bio-Rad, Hercules, CA, USA) using the Trans-Blot Turbo transfer system with Trans-Blot Turbo Transfer Kit reagents (Bio-Rad, Hercules, CA, USA), according to the manufacturer’s instructions. Non-specific binding sites on the membrane were blocked with either Roti-Block solution (Carl Roth, Karlsruhe, Germany) or 5% bovine serum albumin (BSA) in Tris-buffered saline with 0.1% Tween 20 (TBST) (VWR International, Radnor, PA, USA) for one hour at room temperature. The OxPhos Antibody Cocktail and antibodies against ATP synthase subunits (ATP5F1, ATPB, and ATP5G1) were obtained from Abcam (Cambridge, UK). Antibodies against the following proteins were used according to the manufacturers’ datasheets: DRP1 (Elabscience, Houston, TX, USA), Mfn2 (Elabscience, Houston, TX, USA), PINK1 (Cusabio, Houston, TX, USA), Parkin (Abclonal, Woburn, MA, USA), TFAM (Santa Cruz Biotechnology, Dallas, TX, USA), and TUFM (Invitrogen, Waltham, MA, USA). An antibody against GAPDH (Santa Cruz Biotechnology, Dallas, TX, USA) was used as a loading control. Incubation with primary antibodies was performed overnight at 4 °C. This was followed by incubation with appropriate horseradish peroxidase (HRP)-conjugated secondary antibodies (Jackson ImmunoResearch, West Grove, PA, USA) for one hour at room temperature. Chemiluminescent detection of the blots was performed using a ChemiDoc MP imaging system (Bio-Rad, Hercules, CA, USA) with Clarity Western ECL Substrate (Bio-Rad, Hercules, CA, USA). The intensity of the protein bands was quantified by densitometry using Image Lab 6.0 software (Bio-Rad, Hercules, CA, USA).

### 2.16. Real-Time PCR for Mitochondrial DNA Copy Number Quantification

Total DNA was isolated using the ExtractDNA Blood & Cells kit (Eurogen, Moscow, Russia). DNA amplification was performed with the intercalating dye SYBR Green I (Eurogen, Moscow, Russia) on a QuantStudio 5 Real-Time PCR System (Thermo Fisher Scientific, Waltham, MA, USA) according to the manufacturer’s instructions. The oligonucleotide primers were synthesized by CJSC Eurogen and are listed in Table 1. Cells with depleted THP1-ρ^0^ mtDNA were used as a positive control.

### 2.17. Statistical Analysis

Data are presented as mean ± standard deviation (SD). All experiments were performed in at least five independent replicates (*n* ≥ 5). Statistical significance of differences was determined using one-way analysis of variance (ANOVA) followed by the Holm–Sidak multiple comparison test. We utilized the Wald test for evaluating differential gene expression and permutation test for GSEA, incorporating Benjamini–Hochberg false discovery rate (FDR) correction. A *p*-value of less than 0.05 (*p* < 0.05) or false discovery rate less than 0.05 (FDR < 0.05) was considered statistically significant. The magnitude of the observed effects was estimated using the standardized mean difference (Hedges’ g), calculated with the Pingouin library (version 0.5.5). A Hedges’s value greater than 0.8 indicates a large effect size (Table S1) [40].

## 3. Results

### 3.1. Transcriptomic Profiling of Mitochondrial Pathways and Metabolism-Related Genes in THP-1 Acute Myeloid Leukemia Cells During Aseptic Pro-Inflammatory Activation

Total RNA sequencing was performed to evaluate the transcriptional activity of mitochondrial pathways (MP) and mitochondrial metabolism-related genes (MMRG) in apoptosis-resistant THP-1 cells grown in three-dimensional high-density cultures (THP-1HDC), compared to apoptosis-sensitive THP-1 cells maintained in low-density cultures (THP-1LDC). Principal Component Analysis (PCA) and distance matrix computation were performed for both HDC and LDC conditions to assess sample relationships based on gene expression profiles. Both analyses revealed clear separation between the comparison groups (HDC vs. LDC) alongside strong intra-group similarity, indicating distinct transcriptional signatures between conditions (Figure S1). Transcriptional activity of mitochondrial pathways was analyzed using Gene Set Enrichment Analysis (GSEA) with 149 MitoPathway gene sets from the Human MitoCarta 3.0 database.

**Figure 1:**
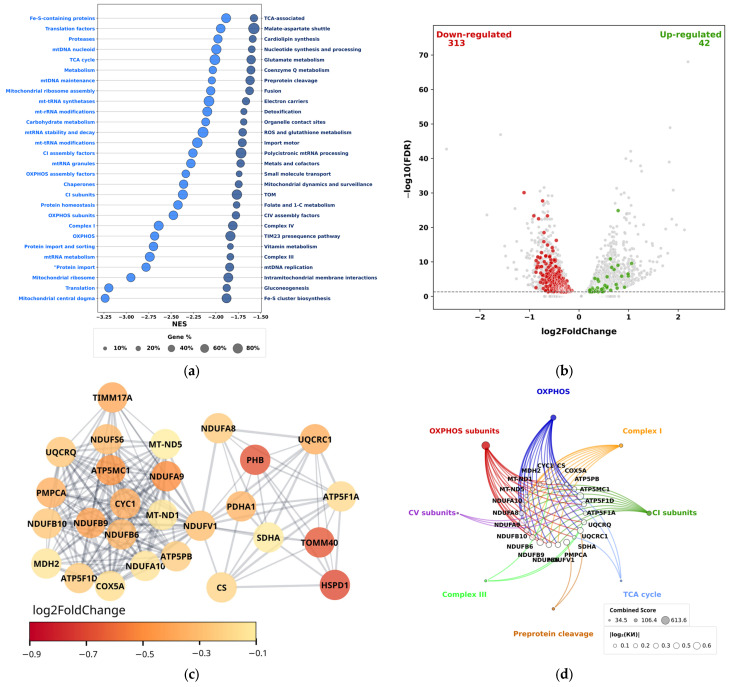
GSEA using MitoPathway gene sets from the Human MitoCarta 3.0 database in THP-1HDC cells compared to THP-1LDC cells (**a**). The distribution of expression of MMRG processes from the Human MitoCarta 3.0 database, showing the expression change index log2FoldChange and the reliability of expression change (-log10(FDR)) (**b**). Interaction network of products of downregulated MMRGs (**c**). Functional annotation of hub genes with reduced expression (**d**). DB—MitoPathways, FDR < 0.05.

We found that in apoptosis-resistant THP-1HDC cells, relative to THP-1LDC cells, 56 gene sets had a negative normalized enrichment score (NES), while the remaining 93 gene sets did not show significant changes (FDR ≥ 0.05). Among the identified gene sets, those with the most negative NES values were “Mitochondrial central dogma”, “Translation”, “Mitochondrial ribosome”, “Protein import”, “mtRNA metabolism”, “Protein import and sorting”, “OXPHOS”, “Complex I”, “OXPHOS subunits”, and “Protein homeostasis” (Figure 1a).

When analyzing the differential expression of MMRG in THP-1HDC AML cells, we identified 355 genes (313 downregulated and 42 upregulated) with significant (FDR < 0.05) expression changes relative to THP-1LDC cells (Figure 1b, Table S2).

A network analysis of protein–protein interactions (PPI) for differentially expressed MMRG was performed using the Cytoscape software and the STRINGdb database. For genes with reduced expression, 304 genes formed a single interaction network. Clustering of the interaction networks for downregulated MMRG products identified 21 hub genes encoding subunits of Complex I (*NDUFS6*, *NDUFA9*, *NDUFB10*, *NDUFB9*, *NDUFB6*, *NDUFA8*, *NDUFA10*, *NDUFV1*, *MT-ND1*, *MT-ND5* genes), Complex III (*UQCRQ*, *UQCRC1*, *CYC1* genes), Complex IV (*COX5A* gene), and Complex V (ATP synthase) (*ATP5MC1*, *ATP5PB*, *ATP5F1A*, *ATP5F1D*, *ATP5F1O* genes) of the respiratory chain; enzymes of the tricarboxylic acid cycle: citrate synthase (*CS* gene), malate dehydrogenase 2 (*MDH2* gene), and succinate dehydrogenase (*SDHA* gene); the E1-α subunit of the pyruvate dehydrogenase complex (*PDHA1* gene); proteins involved in mitochondrial protein transport: a component of the TIM23 complex (*TIMM17A* gene) and the mitochondrial outer membrane porin (*TOMM40* gene); the mitochondrial chaperone HSP60 (*HSPD1* gene) and the prohibitin protein (*PHB* gene) (Figure 1c). Among the identified genes, the most interesting are *NDUFS6*, *NDUFA9*, *MT-ND5*, *SDHA*, *NDUFB10*, *NDUFB9*, *NDUFB6*, *NDUFA8*, *NDUFA10*, *MDH2*, *CS*, *PDHA1*, *TIMM17A*, *TOMM40*, *HSPD1*, *PHB*, *CYC1*, and *COX5A*, as their products have been shown to be involved in regulating drug resistance in tumor cells and/or the antitumor immune response, and may also be associated with the activation of inflammatory signaling pathways [41,42,43,44,45,46,47,48,49,50,51,52,53,54,55,56,57,58,59,60,61,62,63,64,65,66,67,68,69,70,71,72,73,74,75].

Next, functional annotation was performed for the downregulated hub MMRGs using the identified downregulated mitochondrial pathways (MPs) and the MitoPathway gene sets from the Human MitoCarta 3.0 database. Among the downregulated MPs, the eight MPs with the highest combined score (a coefficient calculated as activity coefficient × −log10(*p*-value)) were selected. Associations were found for 19 downregulated hub MMRGs with the “OXPHOS” and “OXPHOS subunits” MPs, for 10 gene products with the “Complex I” MP, for 9 gene products with the “CI subunits” MP, for 3 gene products with either the “Complex III” or “TCA cycle” MPs, and for 2 gene products with the “Preprotein cleavage” MP (Figure 1d).

In turn, the upregulated hub MMRGs formed two isolated interaction networks (Figure S2). These networks included 21 genes (comprising 16 and 5 genes, respectively) encoding proteins involved in: fatty acid oxidation (*DBI*, *DECR1*, *ACADS*, *ECHDC2*); fatty acid activation (*ACSF2*); assembly of mitochondrial ribosomes and synthesis of mitochondrial proteins (*NOA1*); GABA metabolism (*ABAT*); propionate and valine metabolism (*ALDH6A1*); transfer of acyl groups in mitochondria (*CRAT*); heme synthesis (*ALAS1* and *PPOX*); lactate metabolism (*LDHD*); processing of mitochondrial proteins (*PMPCB*); ATP/ADP transport across the mitochondrial membrane (*SLC25A6*); insertion of proteins into the inner mitochondrial membrane (*OXA1L*); metabolism of the amino acids lysine, hydroxylysine, and tryptophan (*DHTKD1*); autophagy and degradation of aggregated proteins (*NBR1*); regulation of apoptosis and autophagy (*FKBP8*); induction of mitophagy and apoptosis (*BNIP3L*); immune response and mitochondrial apoptosis (*MAVS*); and the antiapoptotic protein Bcl-2 (*BCL2*) (Figure S2). It has been shown that the upregulated hub MMRG genes *BCL2*, *MAVS*, *FKBP8*, *NBR1*, and *SLC25A6* and their products can participate in regulating drug resistance of tumour cells and the antitumour immune response and may be associated with the activation of inflammatory signalling pathways [76,77,78,79,80,81,82,83,84,85]. However, functional annotation using the MitoPathway cluster revealed no significant associations with upregulated MPs.

Thus, in drug-resistant THP-1 AML cells exhibiting a sterile inflammatory response in three-dimensional high-density cell cultures, a pronounced suppression of transcriptional activity in OXPHOS and mitochondrial biogenesis genes, as well as an increase in the transcriptional activity of genes involved in alternative energy supply pathways (such as fatty acid β-oxidation), was observed. This pattern is characteristic of conditions involving the activation of inflammatory signaling pathways [86,87,88,89,90].

### 3.2. Analysis of Mitochondrial Content and Ultrastructure in THP-1 Acute Myeloid Leukemia Cells During Aseptic Inflammatory Activation

Mitochondrial functional activity is known to correlate with mitochondrial number and size [91]. We assessed mtDNA copy number, mitochondrial mass, and mitochondrial ultrastructure in apoptosis-resistant THP-1HDC cells exhibiting aseptic inflammatory activation. These parameters were compared with those in apoptosis-sensitive THP-1LDC cells and in THP-1 cells treated with lipopolysaccharide (THP-1LPS), which induces mitochondrial dysfunction through activation of inflammatory pathways [92,93]. THP-1LPS cells also demonstrate increased resistance to chemotherapeutic agents [19,94]. Additionally, we included THP-1 cells with depleted mitochondrial DNA (THP-1-ρ^0^), a well-known model of mitochondrial dysfunction [95].

**Figure 2:**
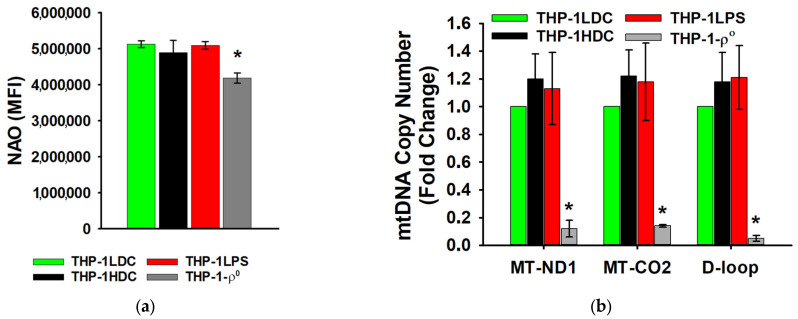
Mitochondrial mass (**a**) and mtDNA copy number (**b**) in THP-1LDC, THP-1HDC, THP-1LPS, and THP-1-ρ^0^ cells. MFI (mean fluorescence intensity; arbitrary units, arb. units) was measured in cells stained with NAO. The data is given as an average value ± SD. *—*p* < 0.05 in comparison with THP-1LDC cells.

In both THP-1HDC and THP-1LPS cells, the relative mitochondrial mass (Figure 2a) and the mtDNA copy number (Figure 2b) did not change significantly compared to THP-1LDC cells. In contrast, THP-1-ρ^0^ cells exhibited a marked decrease in both mtDNA copy number (*p* < 0.01) and relative mitochondrial mass (*p* < 0.05) (Figure 2a,b).

**Figure 3:**
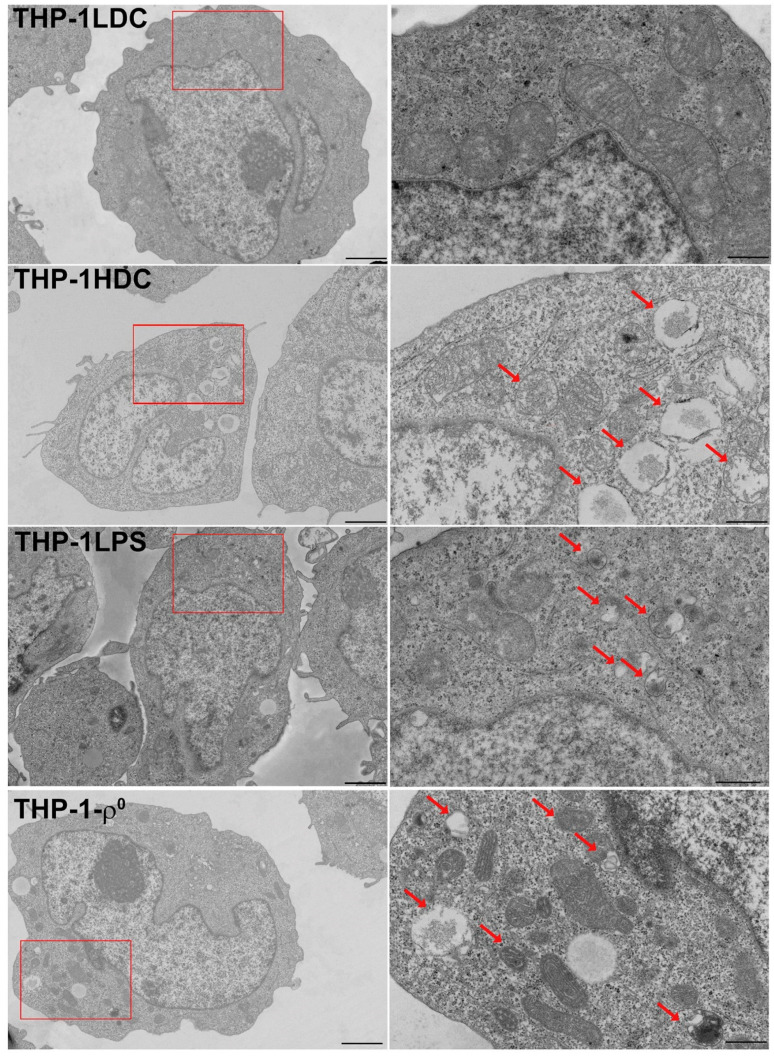
Representative electron micrographs of THP-1LDC, THP-1HDC, THP-1LPS, and THP-1-ρ^0^ cells at low (**left** panels) and high (**right** panels) magnification. The red boxes in the **left** panel indicate the magnification zone shown in the **right** panel. Abnormal mitochondria are indicated by arrows. Scale bars: 1 μm (low magnification) and 600 nm (high magnification). The original electron micrographs are shown in Figure S3.

Electron morphometric analysis revealed that in apoptosis-resistant THP-1HDC and THP-1LPS cells, compared to apoptosis-sensitive THP-1LDC cells, the proportion of medium-sized (0.19–0.49 μm^2^) and small (0.04–0.10 μm^2^) mitochondria increased, while the proportion of large (0.50–0.90 μm^2^) mitochondria decreased (Table 2; Figure 3). In addition, THP-1HDC and THP-1LPS cells contained abnormal mitochondria of medium and small size with structural abnormalities (up to 20% of the total number of mitochondria in a cell), which were absent in THP-1LDC cells (Figure 3).

THP-1-ρ^0^ cells also exhibited an increased proportion of small and medium-sized mitochondria, a decreased proportion of large mitochondria, and the presence of abnormal mitochondria (up to 37% of the total number of mitochondria in a cell) (Table 2, Figure 3), which is consistent with the results of other studies [96,97].

Thus, our results indicate that apoptosis-resistant THP-1HDC cells exhibiting a sterile inflammatory response undergo morphological changes in their mitochondria without a change in mitochondrial content.

### 3.3. Characteristics of Mitochondrial Respiration in THP-1 Acute Myeloid Leukemia Cells During Aseptic Inflammatory Activation

Next, the functional state of mitochondria was compared between apoptosis-resistant THP-1HDC cells and apoptosis-sensitive THP-1LDC cells. Specifically, we measured oxygen consumption in permeabilized, levels of respiratory chain complex proteins and ATP synthase subunits, mitochondrial membrane potential, intracellular ROS production, extracellular lactate concentration, and ADP/ATP and NAD^+^/NADH ratios. THP-1LPS cells served as a control for proinflammatory activation associated with mitochondrial dysfunction.

**Figure 4:**
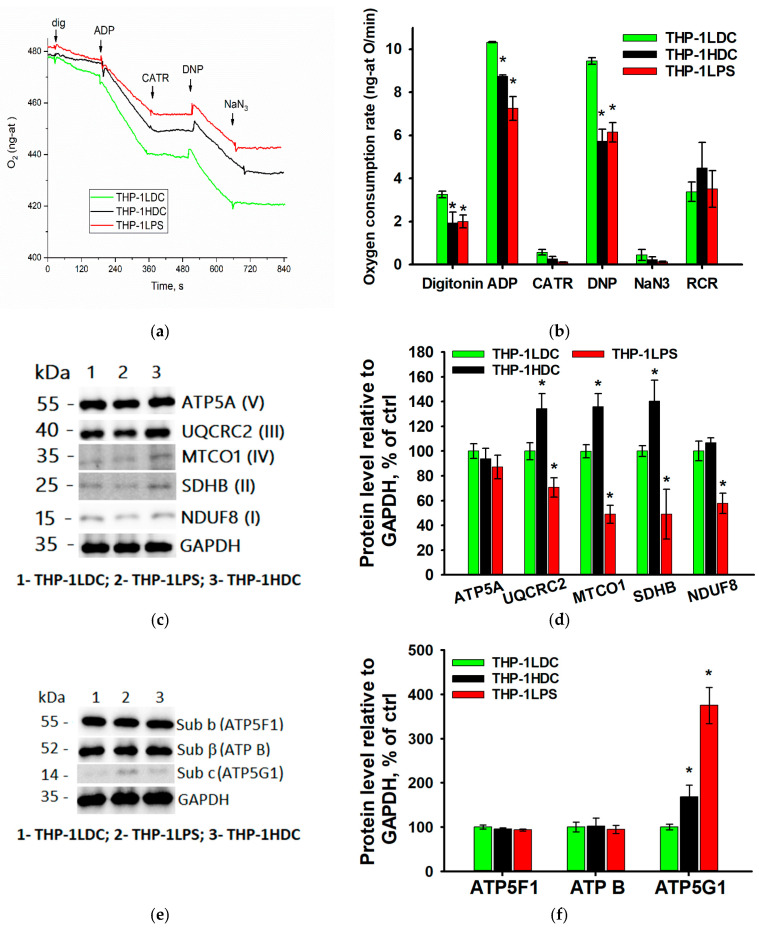
Mitochondrial respiratory chain activity (**a**,**b**), electron transport chain (ETC) protein levels (**c**,**d**), and ATP synthase subunit expression (**e**,**f**) in THP-1LDC, THP-1LPS, and THP-1HDC cells. (**a**,**b**) Representative oxygen consumption curves (**a**) and respiration rates (**b**) in THP-1LDC, THP-1LPS, and THP-1HDC cells oxidizing Complex II substrates. RCR—the respiratory control ratio. Arrows indicate additions of: 0.005% digitonin, 2 mM ADP, 1 µM carboxyatractyloside (CATR), 30 µM 2,4-dinitrophenol (DNP), and 1 mM NaN_3_. (**c**) Representative Western blot of ETC complex proteins in THP-1LDC (column 1), THP-1LPS (column 2), and THP-1HDC (column 3) cells. The original WB images are shown in Figure S4. (**d**) Quantitative analysis of ETC protein levels normalized to GAPDH. (**e**) Representative Western blot of ATP synthase subunits in THP-1LDC (column 1), THP-1LPS (column 2), and THP-1HDC (column 3) cells. The original WB images are shown in Figure S5. (**f**) Quantitative analysis of ATP synthase subunit levels normalized to GAPDH. Data are presented relative to control THP-1LDC cells, set to 100%. The data is given as an average value ± SD. *—*p* < 0.05 in comparison with THP-1LDC cells.

Figure 4a shows representative oxygen electrode traces of cellular respiration supported by the Complex II substrate (succinate in the presence of rotenone). The rate of non-stimulated respiration at rest (basal respiratory rate, State 2) was estimated after adding 0.005% digitonin to the cell suspension and permeabilization of the plasma membrane. Subsequently, after the addition of 2 mM ADP, the rate of oxidative phosphorylation (State 3 respiration) was measured. This process is regulated by ATP synthase activity and/or the mitochondrial ADP/ATP transport system and is inhibited by the addition of 1 µM carboxyatractyloside (CATR), a specific inhibitor of the adenine nucleotide translocase (ANT). The maximum (uncoupled) respiratory rate was measured after addition of the uncoupler 2,4-dinitrophenol (DNP; 30 µM). Non-mitochondrial oxygen consumption was determined following inhibition of cytochrome c oxidase with sodium azide (NaN_3_). The respiratory control ratio (RCR) was calculated to reflect the ratio of active respiration (State 3) to resting respiration (State 2), thereby characterizing the degree of mitochondrial coupling and the efficiency of oxidative phosphorylation.

Figure 4b shows the average respiration rates using a Complex II substrate for THP-1LDC, THP-1HDC, and THP-1LPS cells. The obtained data demonstrate that in resistant THP-1HDC and THP-1LPS cells, compared to THP-1LDC cells, both the resting respiration rate (basal respiration) and the oxidative phosphorylation rate in State 3 significantly decreased. This may indicate decreased activity of ATP synthase and/or the ADP transport system. Additionally, a decrease in the maximum (uncoupled) respiration rate was observed, which may reflect decreased activity of the mitochondrial respiratory chain as a whole (Figure 4a,b). At the same time, the RCR value for THP-1HDC and THP-1LPS cells did not show a statistically significant difference with the indicator for THP-1LDC cells (Figure 4b). This suggests that the efficiency of mitochondrial coupling remains preserved in THP-1HDC cells, meaning the mechanisms coupling substrate oxidation to ATP synthesis function adequately. Similar alterations in mitochondrial respiration activity were also detected in HL-60 AML cells cultured in a three-dimensional high-density system or treated with LPS (Figure S6).

The noted decrease in mitochondrial respiratory chain activity in apoptosis-resistant THP-1HDC and THP-1LPS cells, relative to sensitive cells, was observed with changes in the levels of respiratory chain complexes and ATP synthase subunits (Figure 4e,f). In THP-1HDC cells, the levels of SDHB (a complex II subunit), UQCRC2 (a complex III subunit), and MTCO1 (a complex IV subunit) increased by 30%, whereas the abundance of NDUFB8 (a complex I subunit) and ATP5A (a complex V subunit) remained unchanged compared to THP-1LDC cells. The levels of NDUFB8, SDHB, UQCRC2, and MTCO1 were markedly decreased in THP-1LPS cells. Additionally, it was shown that neither THP-1HDC nor THP-1LPS cells showed statistically significant changes in the levels of the α (catalytic domain F1, ATP5F1) and β (Fo transmembrane domain, ATP5B) subunits of mitochondrial ATPase. Nevertheless, the level of the c subunit of the ATPase transmembrane domain (ATP5G1) was elevated compared to apoptosis-sensitive THP-1LDC cells (Figure 4e,f), which may be due to its impaired processing [38].

**Figure 5:**
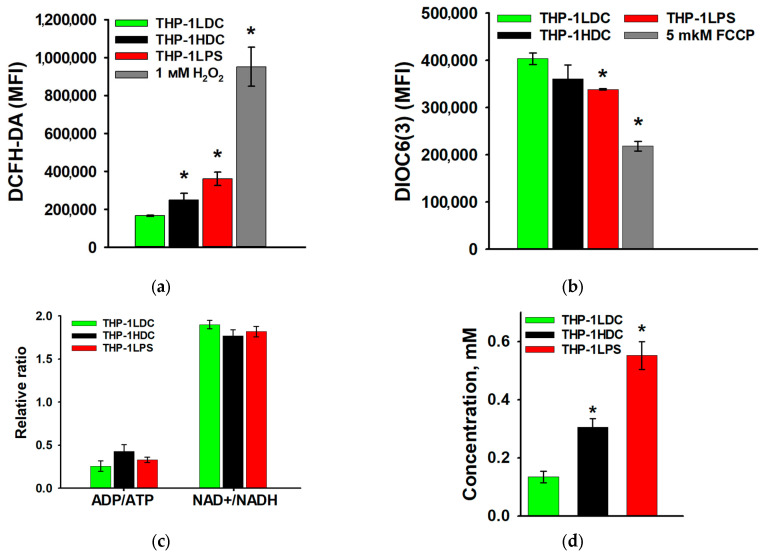
Intracellular ROS production (**a**), mitochondrial membrane potential (**b**), ADP/ATP and NAD +/NADH ratios (**c**), and lactate concentration in the culture medium (**d**) of THP-1LDC, THP-1LPS, and THP-1HDC cells. MFI (mean fluorescence intensity) was measured in cells loaded with DCFH-DA or DiOC6(3). The data is given as an average value ± SD. *—*p* < 0.05 in comparison with THP-1LDC cells.

Furthermore, it was found that intracellular ROS production increased in apoptosis-resistant THP-1HDC and THP-1LPS cells compared to apoptosis-sensitive THP-1LDC cells (Figure 5a), while the mitochondrial membrane potential decreased (Figure 5b). Analysis of the ADP/ATP and NAD^+^/NADH ratios revealed no statistically significant changes in resistant THP-1HDC and THP-1LPS cells compared to sensitive THP-1LDC cells (Figure 5c). However, apoptosis-resistant THP-1HDC and THP-1LPS cells accumulated more lactate in the culture medium compared to sensitive THP-1LDC cells, with values rising from 0.13 ± 0.02 mM (THP-1LDC) to 0.30 ± 0.03 mM (THP-1HDC) and 0.55 ± 0.05 mM (THP-1LPS) (Figure 5d). A decrease in mitochondrial membrane potential and an increase in ROS production were also observed in MV4-11 AML cells cultured in three-dimensional high-density cultures (Figure S7). In contrast, HL-60 cells under the same culture conditions showed only an increase in ROS production (Figure S7).

Thus, the obtained results indicate that in apoptosis-resistant THP-1 HDC cells with aseptic pro-inflammatory activation, mitochondrial respiratory activity is decreased. This occurs against a background of changes in the content of subunits of respiratory chain complexes and ATP synthase, as well as an increase in intracellular ROS and accumulation of lactate in the culture medium.

### 3.4. Key Marker Proteins of Mitochondrial Quality Control System in THP-1 Acute Myeloid Leukemia Cells During Aseptic Inflammatory Activation

Mitochondrial quality control (MQC) is a complex system of processes that work together to protect mitochondria from harm and prevent the buildup of damaged mitochondria. These processes primarily rely on three distinct mechanisms: mitochondrial biogenesis, dynamics, and mitophagy.

Mitochondrial dynamics (fusion and fission) and mitophagy are critically important for mitochondrial function [98]. Fusion enables the exchange of metabolites, proteins, and mitochondrial DNA between organelles, facilitating complementation of damaged mitochondria [98]. Fission increases mitochondrial number and ensures their proper distribution to daughter cells during cell division [98]. Mitophagy is a selective process that removes excess or damaged mitochondria, thereby optimizing energy metabolism [98]. We therefore assessed the levels of key mitochondrial regulatory proteins (TFAM, TUFM, PINK1, Parkin, MFN2, and DRP1) in apoptosis-resistant THP-1HDC cells compared to sensitive THP-1LDC cells. THP-1LPS cells served as a control for proinflammatory cellular activation.

**Figure 6:**
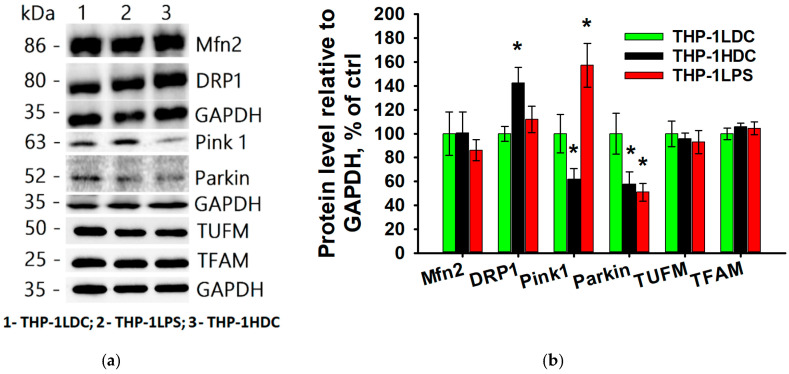
Levels of key mitochondrial regulatory proteins (Tfam, Tufm, Pink1, Parkin, Mfn2, and DRP1) in THP-1LDC (column 1), THP-1LPS (column 2), and THP-1HDC (column 3) cells. (**a**) Representative Western blot images of TFAM, TUFM, Pink1, Parkin, Mfn2, and DRP1. The original WB images are shown in Figure S8. (**b**) Quantitative analysis of protein levels normalized to GAPDH. Data are presented relative to the control group (THP-1LDC), which was set to 100%. The data is given as an average value ± SD. *—*p* < 0.05 in comparison with THP-1LDC cells.

Figure 6 shows that the levels of TFAM and TUFM proteins, which regulate mitochondrial biogenesis [99,100], and the Mfn2 protein, which regulates mitochondrial fusion [101], did not change significantly in either THP-1HDC or THP-1LPS cells compared to THP-1LDC cells. In contrast, the levels of Pink1 and Parkin proteins, involved in regulating mitochondrial protein quality control and mitophagy [102], were altered in the resistant THP-1HDC and THP-1LPS cells compared to the THP-1LDC cells (Figure 6). Specifically, the Parkin protein level decreased by 50% in both THP-1HDC and THP-1LPS cells compared to THP-1LDC cells (Figure 6). The Pink1 protein level decreased by 40% in THP-1HDC cells but increased by 50% in THP-1LPS cells compared to THP-1LDC cells (Figure 6). Furthermore, THP-1HDC cells—but not THP-1LPS cells—were characterized by a 40% increase in the level of DRP1 protein, which regulates mitochondrial fission [103], compared to THP-1LDC cells (Figure 6).

### 3.5. Navitoclax Sensitizes Aseptically Inflammatory Activated AML Cells to Etoposide and TRAIL-Induced Apoptosis

**Figure 7:**
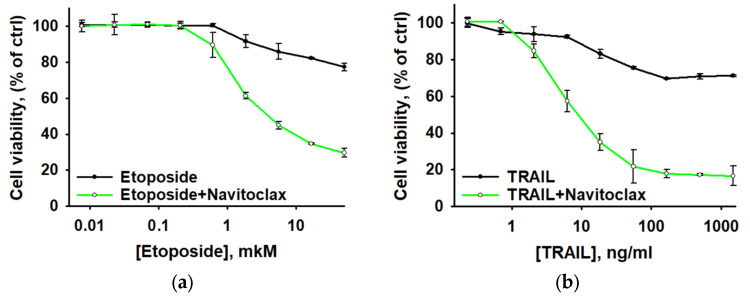
Navitoclax Reduces THP-1HDC Cell Resistance to Etoposide (**a**) and TRAIL (**b**). The data is given as an average value ± SD.

As noted above, the *BCL2* gene was identified among the upregulated hub MMRGs in apoptosis-resistant THP-1HDC cells. The product of this gene, the antiapoptotic protein Bcl-2, is a key regulator of tumor cell apoptosis [104]. Furthermore, our previous work showed significantly increased levels of the anti-apoptotic protein Bcl-2 in THP-1HDC cells compared with THP-1LDC cells [19]. In this regard, further in the work, to assess the possibility of overcoming the resistance of THP-1 AML cells in high-density cultures, we used a low-molecular-weight inhibitor of Bcl-2 family proteins (Bcl-2, Bcl-w and Bcl-xL)—BH3 mimetic Navitoclax (ABT-263). Pre-incubation of AML cells in a high-density culture with Navitoclax (1 µM, 4 h) resulted in a decrease in the population of resistant cells. The percentage of cells resistant to a maximum concentration of etoposide (50 µM) diminished from 77 ± 2% to 30 ± 2%, while the percentage resistant to recombinant TRAIL protein (1 mg/mL) decreased from 71 ± 2% to 17 ± 5% (Figure 7). Similar results were obtained for HL-60 and MV4-11 cells (Figure S9).

## 4. Discussion

The role of mitochondria in the vital processes of AML cells is not limited to their energy function but is also key in regulating the balance between sensitivity and resistance to apoptosis [4,5,7,8,9]. In addition, mitochondria are among the most important regulators of the aseptic inflammatory response, which is associated with increased survival and drug resistance in AML cells [9,10,11,14,15]. Therefore, in this study, we focused on investigating key mitochondrial functions in apoptosis-resistant AML cells using three-dimensional high-density cell cultures—a simple and reproducible in vitro model of a sterile proinflammatory response.

The results of transcriptomic profiling revealed a significant decrease in the transcriptional activity of genes associated with oxidative phosphorylation and the tricarboxylic acid cycle in apoptosis-resistant THP-1 AML cells under conditions of a sterile proinflammatory response in three-dimensional high-density cultures. This pattern is characteristic of activated inflammatory signaling pathways [86,87,88].

Under conditions of both aseptic pro-inflammatory activation in three-dimensional high-density cultures and LPS treatment, a significant decrease in mitochondrial respiration rate was observed. This reduction was accompanied by maintained mitochondrial coupling, along with increased production of reactive oxygen species (ROS) and lactate accumulation in the culture medium. Furthermore, under conditions of aseptic pro-inflammatory activation, an increase in the content of subunits II, III, and IV of the respiratory complexes, as well as subunit C of mitochondrial ATP synthase, and a slight decrease in mitochondrial membrane potential were detected. These changes may be attributed to the preservation of partial oxidative phosphorylation activity and an incomplete shift towards glycolysis or beta-oxidation of fatty acids, which requires further investigation. In cells treated with LPS (modeling septic proinflammatory activation), we observed a decrease in the content of subunits of all mitochondrial respiratory complexes and a reduction in mitochondrial membrane potential. These changes, along with high lactate production, suggest a global shift in cell metabolism from oxidative phosphorylation to aerobic glycolysis, which is characteristic of LPS-induced proinflammatory activation [25,26].

It should be noted that in HL-60 cells, as in THP-1 cells, the activity of mitochondrial respiration decreased and the production of intracellular reactive oxygen species increased under HDC conditions. At the same time, MV4-11 cells under HDC conditions, on the contrary, did not show a decrease in respiration and reduced the production of intracellular ROS. We suggest this is because, unlike the HL-60 and THP-1 cell lines, MV4-11 cells harbor the FLT3-ITD mutation [105], which causes constitutive activation of FLT3-associated signaling pathways, including PI3K/Akt, Ras/MEK/MAPK, and STAT3/5 [106,107]. Several studies have demonstrated that increased OXPHOS activity is a key metabolic feature of FLT3-ITD-positive AML cells [108,109,110], which contributes to their survival and proliferation. On the other hand, MV4-11 cells can use alternative adaptive mechanisms, including more reliable antioxidant defense systems (glutathione, thioredoxin, catalase, and superoxide dismutase), which allow them to maintain oxidative phosphorylation while reducing ROS levels [111]. However, this mechanism requires further research. Such differential metabolic adaptation may be important for the development of personalized therapeutic strategies tailored to specific subtypes of AML.

Furthermore, in apoptosis-resistant THP-1 AML cells exhibiting a sterile inflammatory response, we observed an increase in the level of dynamin-1-like protein (DRP1)—a key GTPase regulating mitochondrial division—and an increase in the proportion of small and medium-sized mitochondria [103]. In contrast, no changes were detected in the level of mitofusin-2 (Mfn2), a key GTPase regulating mitochondrial fusion [101]. These data likely indicate a shift in the balance of mitochondrial dynamics toward fission, a process known to occur during M1 macrophage polarization in the inflammatory response [112]. Conversely, in LPS-treated cells, we also observed an increase in the proportion of small and medium-sized mitochondria; however, the level of DRP1 protein remained unchanged. This discrepancy may be explained by post-translational modifications of DRP1 (e.g., phosphorylation at Ser616 or dephosphorylation at Ser637), mediated by kinases activated during septic inflammation, which enhance DRP1 activity and stimulate mitochondrial fission without altering its total protein level [113,114].

An interesting observation is that apoptosis-resistant THP-1 AML cells exhibiting a sterile inflammatory response showed a significant decrease in the levels of PINK1 and Parkin, key regulators of mitophagy [102], along with the appearance of abnormal mitochondria. In addition, we found a significant decrease in the expression of the *PHB* gene encoding the protein prohibitin in THP-1HDC cells compared to THP-1LDC cells. It is known that prohibitin plays a key role in mitochondrial quality control, including the process of their selective autophagy [115]. These data suggest that in THP-1 AML cells exhibiting a sterile inflammatory response, the regulation of mitophagy is altered, a phenomenon also observed during the development of an inflammatory response [116].

Moreover, the results of our study demonstrate that the use of a low-molecular-weight inhibitor of antiapoptotic Bcl-2 proteins, such as Navitoclax [117], can increase the sensitivity of apoptosis-resistant THP-1 AML cells to chemotherapeutic agents and the cytokine TRAIL—an effector of antitumor immunity—under conditions of a sterile inflammatory response. These findings indicate the potential for modulating the apoptosis-resistant phenotype in THP-1 AML cells under these conditions.

Thus, in an in vitro model of aseptic pro-inflammatory activation, AML cells exhibit mitochondrial dysfunction. However, unlike the pro-inflammatory activation induced by LPS, this shift did not result in a fully pronounced glycolytic phenotype. This state can be used by AML cells both for the more efficient synthesis of energy substrates (ATP and NADH) and for maintaining redox balance. In both cases, this is essential for ensuring cell viability under stress-like conditions, such as inflammation. We propose that in our in vitro model, a shift in the cytokine profile—specifically, an increased role of proinflammatory cytokines from the TNF family—is a key driver behind the formation of the inflammatory phenotype and its consequent alterations in mitochondrial function. Currently, numerous studies have demonstrated the direct involvement of TNF-α not only in enhancing resistance to cell death but also in suppressing mitochondrial functions, including mitophagy, in tumor cells [118,119]. In our previous studies, which used the same in vitro model, we demonstrated that AML cells under HDC conditions increase the production of pro-inflammatory cytokines TNF-α, IL-1β, IL-6, and IL-9, which also have anti-apoptotic effects [19], as well as activate interferon signaling pathways [22], which is associated with the release of mitochondrial DNA (mtDNA) and activation of the cGAS-STING pathway [120,121]. It is assumed that mtDNA released from dysfunctional mitochondria with impaired mitophagy serves as a pathogen-associated molecular pattern (PAMP) that enhances the pro-inflammatory response through the cGAS-STING-IRF3 pathway and creates a positive feedback loop that supports the inflammatory microenvironment and apoptosis-resistant phenotype.

**Figure 8:**
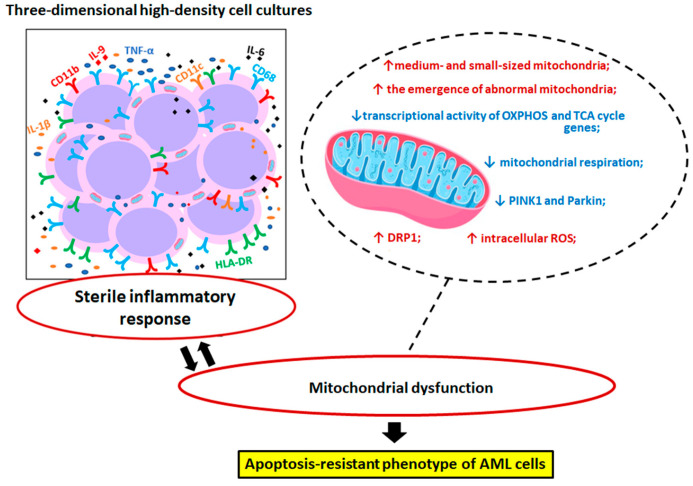
Changes in the mitochondrial status of apoptosis-resistant AML cells under aseptic pro-inflammatory activation in three-dimensional high-density cell cultures. AML—acute myeloid leukemia; OXPHOS—oxidative phosphorylation; TCA—tricarboxylic acid cycle; ROS—reactive oxygen species.

In summary, Figure 8 illustrates the changes in mitochondrial status in apoptosis-resistant acute myeloid leukemia cells undergoing sterile proinflammatory activation in three-dimensional high-density cultures. Considering that sterile inflammation is an integral characteristic of the bone marrow microenvironment in acute myeloid leukemia [1,13,122], simple and reproducible in vitro models—such as three-dimensional, high-density cell cultures in which AML cells exhibit a sterile inflammatory response—are promising tools for studying energy metabolism and anti-apoptotic mechanisms in leukemic cells.

## 5. Conclusions

In apoptosis-resistant AML cells under conditions of aseptic inflammation modeled in three-dimensional high-density cultures, the transcriptional activity of genes associated with oxidative phosphorylation and the tricarboxylic acid cycle was significantly reduced. Additionally, decreased mitochondrial respiration activity and reduced levels of the mitophagy-regulatory proteins PINK1 and Parkin were observed, along with the appearance of pathological mitochondrial forms. In contrast, we found an increase in intracellular reactive oxygen species production, lactate accumulation in the culture medium, and the level of DRP1, as well as an increased proportion of small and medium-sized mitochondria. The obtained data demonstrate that aseptic pro-inflammatory activation leads to metabolic remodeling in AML cells, which combines features of mitochondrial dysfunction. This state may promote the survival of leukemic cells under inflammatory stress and likely mediates the formation of an apoptosis-resistant phenotype.

## Figures and Tables

**Table 1:** Oligonucleotide primers used for quantification of mitochondrial DNA copy number by real-time PCR.

Primer Name	Sequence (5′→3′)
MT-ND-1	F: CACCCAAGAACAGGGTTTGT R: TGGCCATGGGTATGTTGTTAA
MT-CO2	F: AATCGAGTAGTACTCCCGATTG R: TTCTAGGACGATGGGCATGAAA
D-loop	F: CTATCACCCTATTAACCACTCA R: TTCGCCTGTAATATTGAACGTA
18S	F: CTACCACATCCAAGGAAGCA R: TTTTTCGTCACTACCTCCCCG

**Table 2:** Morphometric characteristics of mitochondria in THP-1LDC, THP-1HDC, THP-1LPS, and THP-1-ρ^0^ cells.

	% of Normal Mitochondria per Cell			% of Abnormal Mitochondria per Cell		
	Small	Medium	Large	Small	Medium	Large
THP-1LDC	13.5 ± 8.4	30.2 ± 5.5	56.3 ± 13	-	-	-
THP-1HDC	7.0 ± 4.6	45.8 ± 14.6	13.5 ± 6.2	18.6 ± 7.8	-	8.5 ± 5.9
THP-1LPS	14.9 ± 5.2	45.1 ± 3.8	24.2 ± 3.2	7.0 ± 1.7	5.6 ± 3.7	3.1 ± 1.8
THP-1-ρ^0^	19.6 ± 7.8	29.2 ± 8.9	13.4 ± 2.9	9.1 ± 2.3	11.4 ± 5.4	17.2 ± 10.0

## Data Availability

The RNA-seq data for the cell lines described in this study are available through the Gene Expression Omnibus (GEO) database under accession number GSE298747 or via the direct link: https://www.ncbi.nlm.nih.gov/geo/query/acc.cgi?acc=GSE298747, accessed on 9 June 2025.

## Supplementary Materials

The following supporting information can be downloaded at https://www.mdpi.com/article/10.3390/biom15121635/s1, Figure S1: Principal Component Analysis (PCA) based on all differentially expressed genes (a) shows clear separation between HDC and LDC samples. Each point represents an individual sample. Hierarchical clustering and a heatmap (b), generated from pairwise Euclidean distances between samples, demonstrate both intra-group similarities and clear segregation between the two conditions. The dendrogram and color intensity illustrate sample relatedness; Figure S2: Network of interactions for products of mitochondrial metabolism-related genes upregulated in THP-1HDC cells. DB—Reactome, FDR < 0.05; Figure S3: The original electron micrographs of Figure 3; Figure S4: The original WB images of Figure 4c; Figure S5: The original WB images of Figure 4e; Figure S6: Mitochondrial respiratory chain activity in HL-60 (a) and MV4-11 (b) cells cultured under low-density conditions (LDC), in three-dimensional high-density cultures (HDC), or treated with LPS. The data is given as an average value ± SD. *—*p* < 0.05 in comparison with HL-60LDC or MV4-11LDC cells, respectively; Figure S7: Intracellular ROS production (a,c) and mitochondrial potential (b,d) of HL-60 and MV4-11 cells cultured in low-density (LDC), three-dimensional high-density cultures (HDC) or after LPS (LPS) treatment. MFI is the mean fluorescence intensity of cells loaded with DCFH-DA or DIOC6(3). The data is given as an average value ± SD. *—*p* < 0.05 in comparison with HL-60LDC or MV4-11LDC cells, respectively; Figure S8: The original WB images of Figure 6a; Figure S9: Navitoclax Reduces the Resistance of HL-60 and MV4-11 Cells in the HDC to Etoposide (a,c) and TRAIL (b,d). The data is given as an average value ± SD; Table S1: Effect size matrices across multiple measured parameters; Table S2: MMRGs with differential expression in THP-1HDC AML cells compared to THP-1LDC cells (FDR < 0.05).

## Abbreviations

The following abbreviations are used in this manuscript:

AML	Acute myeloid leukemia
CATR	Carboxyatractyloside
DNP	2,4-dinitrophenol
ETC	Electron transport chain
HDC	High-density cultures
IL	Interleukin
LDC	Low-density cultures
LPS	Lipopolysaccharide
MP	Mitochondrial pathways
MMRG	Mitochondrial metabolism-related genes
ROS	Reactive oxygen species
