# Supplementary material for: Mitochondrial Dysfunction in Apoptosis-Resistant Acute Myeloid Leukemia Cells During a Sterile Inflammatory Response
Source: Biomolecules. 2025 Nov 21;15(12):1635. doi: 10.3390/biom15121635 (PMC12730306; doi:10.3390/biom15121635)
Supplement: Supplementary file 1 [file biomolecules-15-01635-s001.zip › biomolecules-3920965-supplementary.pdf]

Supplementary figures

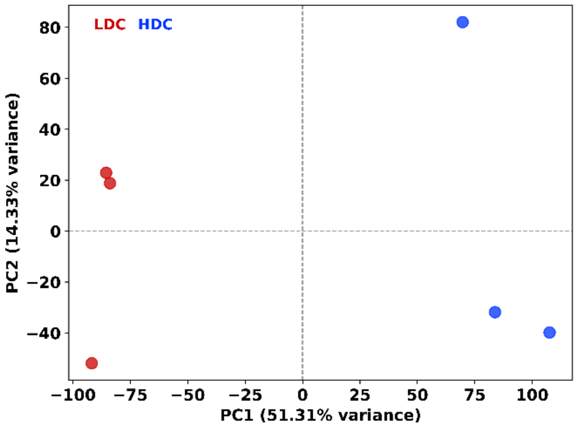

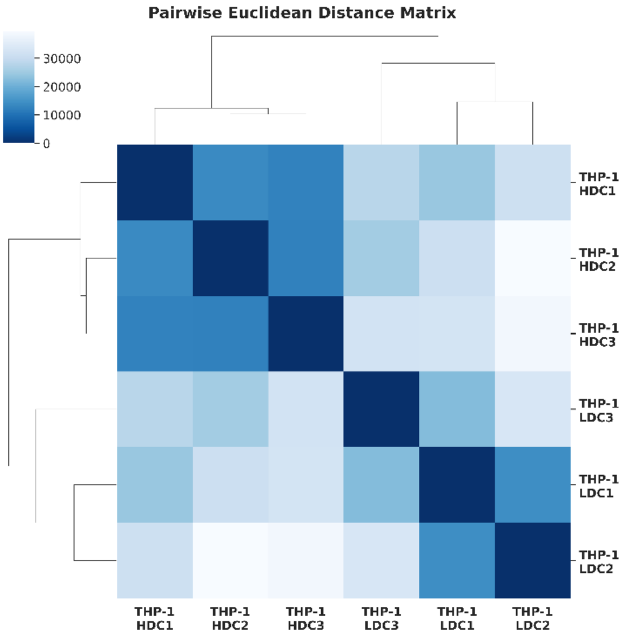

(**a**) (**b**)

Figure S1. Principal Component Analysis (PCA) based on all differentially expressed genes (a) shows clear separation between HDC and LDC samples. Each point represents an individual sample. Hierarchical clustering and a heatmap (b), generated from pairwise Euclidean distances between samples, demonstrate both intra-group similarities and clear segregation between the two conditions. The dendrogram and color intensity illustrate sample relatedness.

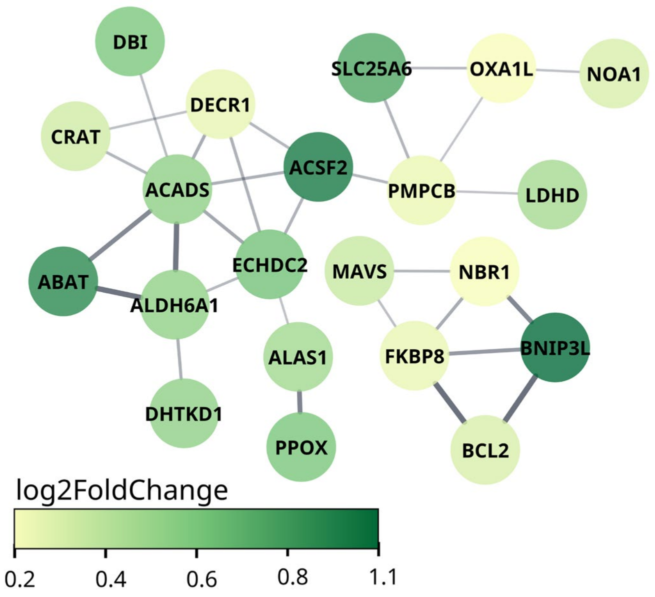

Figure S2. Network of interactions for products of mitochondrial metabolism-related genes upregulated in THP-1HDC cells. DB – Reactome, FDR<0.05.

THP-1LDC

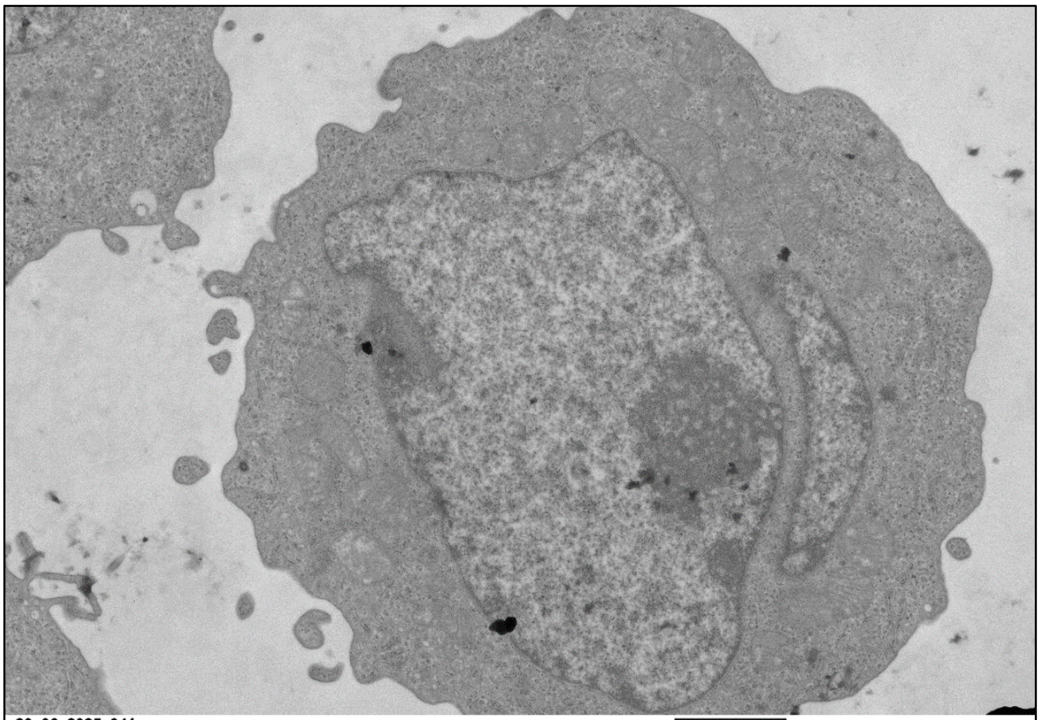

20_06_2025_041
Kontrol
A-1
Calibration: 0.002325 µm/pix
12:12 2025-06-20
Microscopist: Mikheeva
Camera: NS43, Exposure: 500 (ms) x 5 drift frames, Gain: 10, Bin: 1
Gamma: 1.00, No Sharpening, Normal Contrast

2 µm
Accel. Voltage = 80kV
Direct Mag: 3000 x

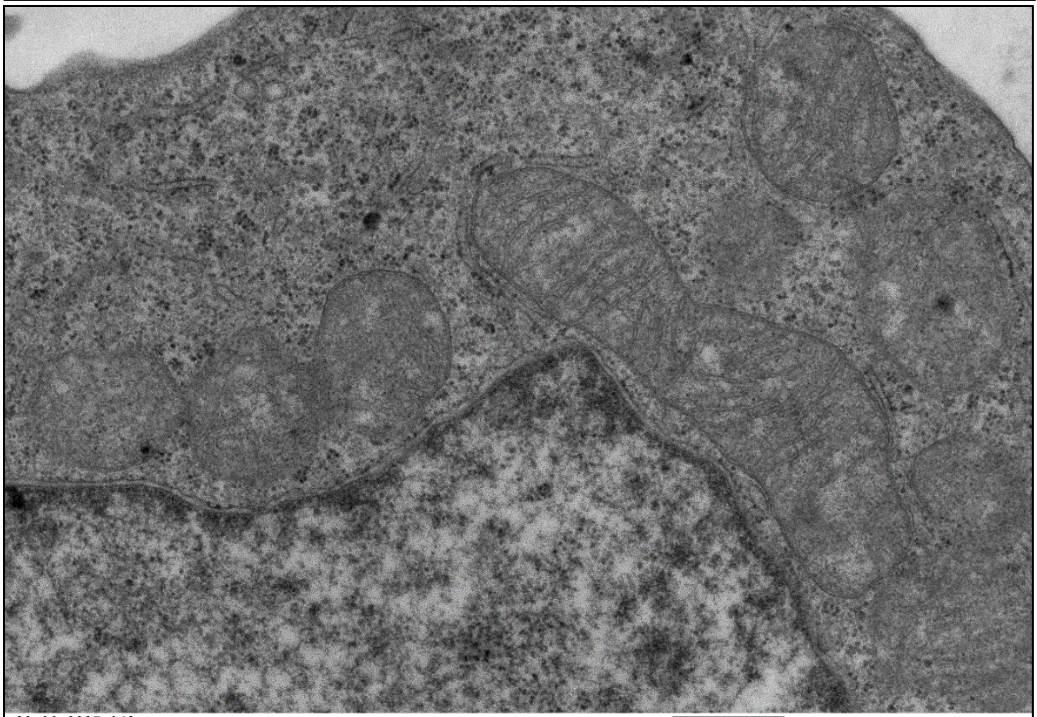

20_06_2025_042
Kontrol
A-1
Calibration: 0.000697 µm/pix
12:14 2025-06-20
Microscopist: Mikheeva
Camera: NS43, Exposure: 500 (ms) x 5 drift frames, Gain: 10, Bin: 1
Gamma: 1.00, No Sharpening, Normal Contrast

600 nm
Accel. Voltage = 80kV
Direct Mag: 10000 x

THP-1HDC

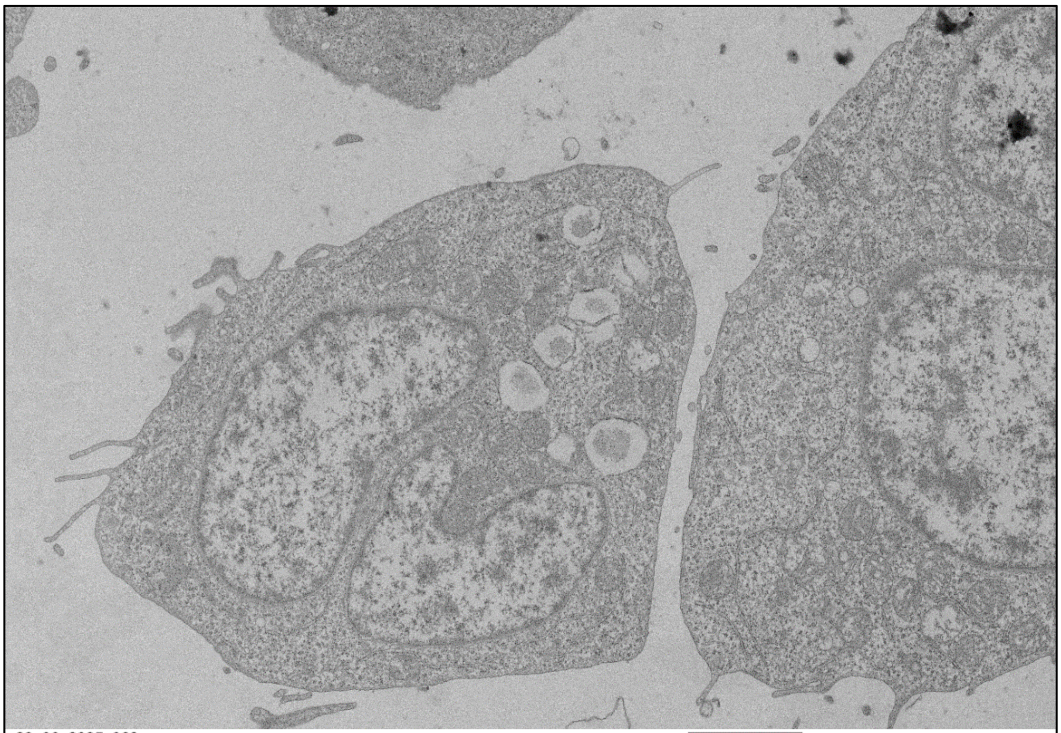

23_06_2025_068
ВПК
C-1
Calibration: 0.002325 µm/pix
13:53 2025-06-23
Microscopist: Mikheeva
Camera: NS43, Exposure: 500 (ms) x 5 drift frames, Gain: 10, Bin: 1
Gamma: 1.00, No Sharpening, Normal Contrast

2 µm
Accel. Voltage = 80kV
Direct Mag: 3000 x

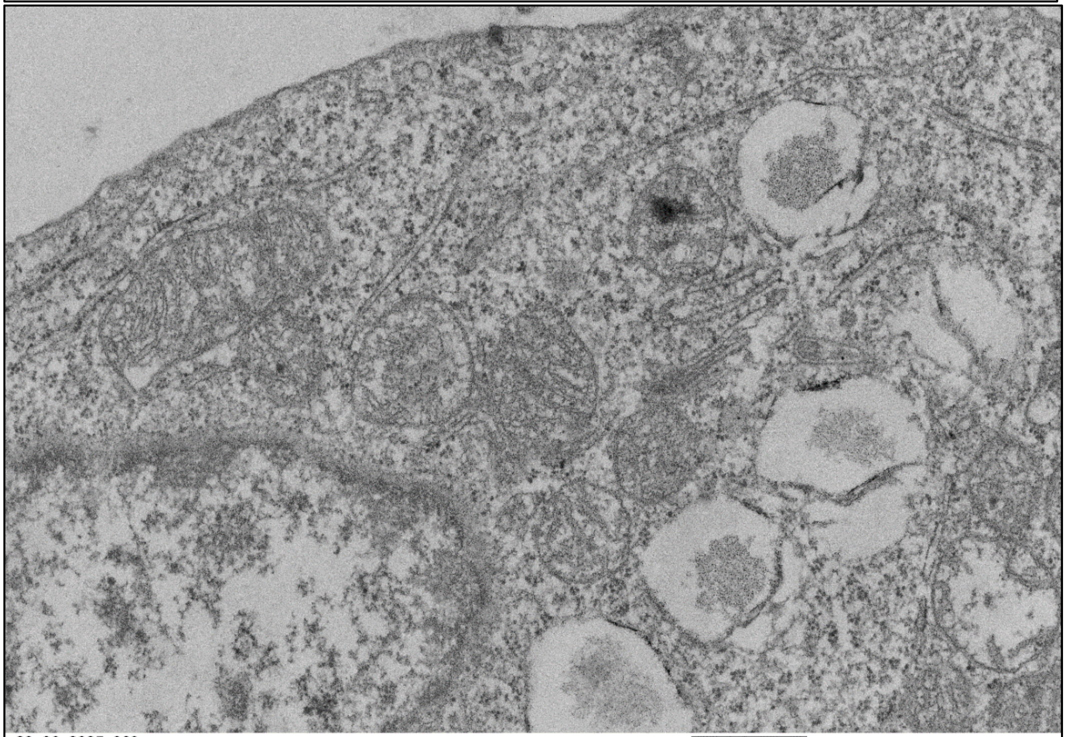

23_06_2025_069
ВПК
C-1
Calibration: 0.000697 µm/pix
13:55 2025-06-23
Microscopist: Mikheeva
Camera: NS43, Exposure: 500 (ms) x 5 drift frames, Gain: 10, Bin: 1
Gamma: 1.00, No Sharpening, Normal Contrast

600 nm
Accel. Voltage = 80kV
Direct Mag: 10000 x

THP-1LPS

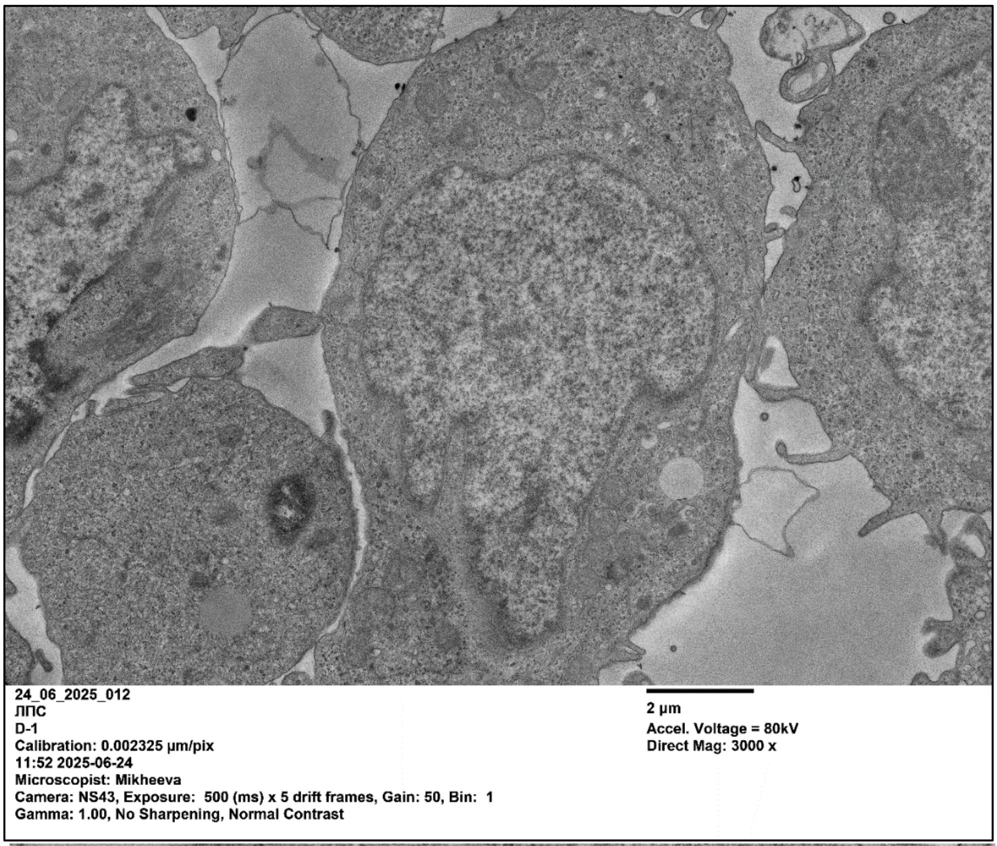
24_06_2025_012
ЛПС
D-1
Calibration: 0.002325 µm/pix
11:52 2025-06-24
Microscopist: Mikheeva
Camera: NS43, Exposure: 500 (ms) x 5 drift frames, Gain: 50, Bin: 1
Gamma: 1.00, No Sharpening, Normal Contrast
2 µm
Accel. Voltage = 80kV
Direct Mag: 3000 x

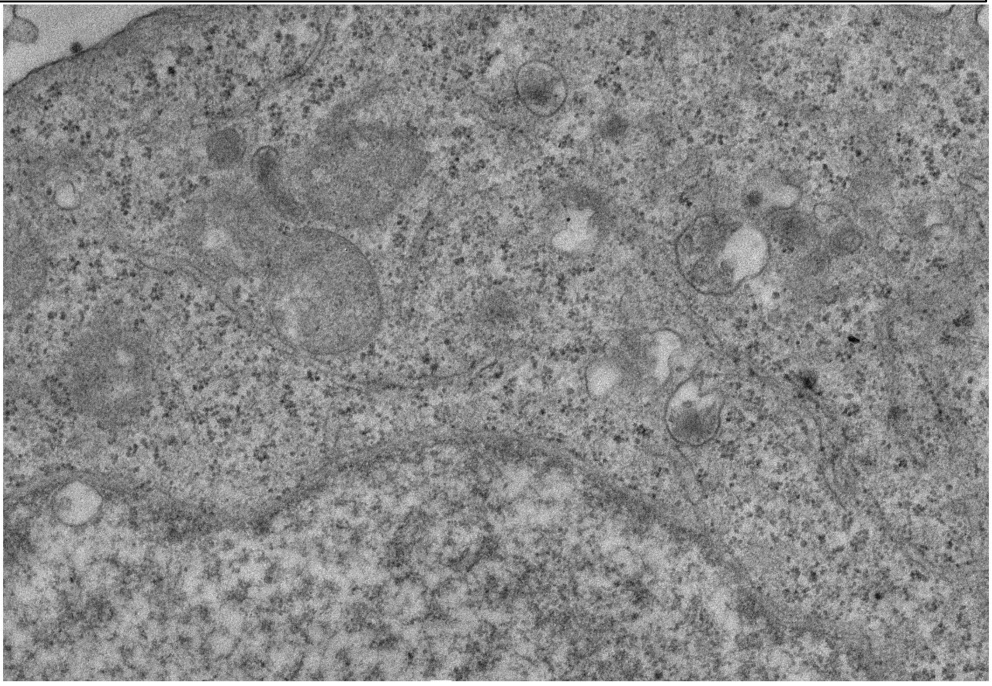

THP-1-po

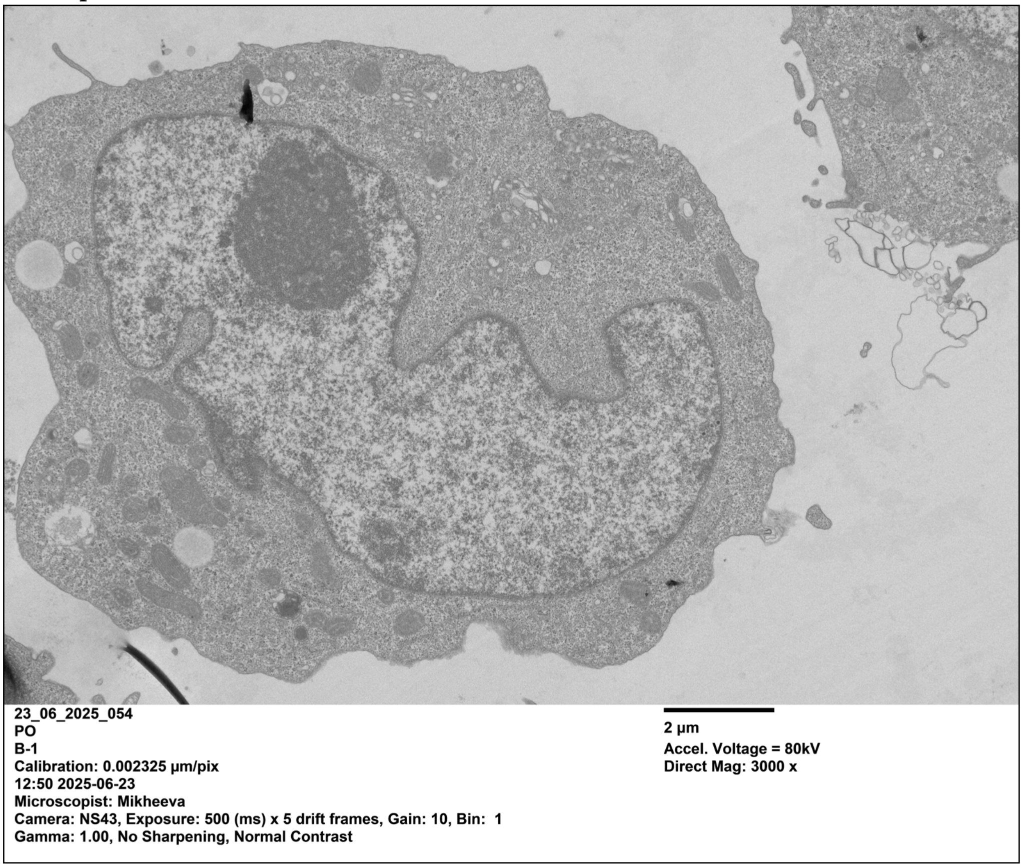

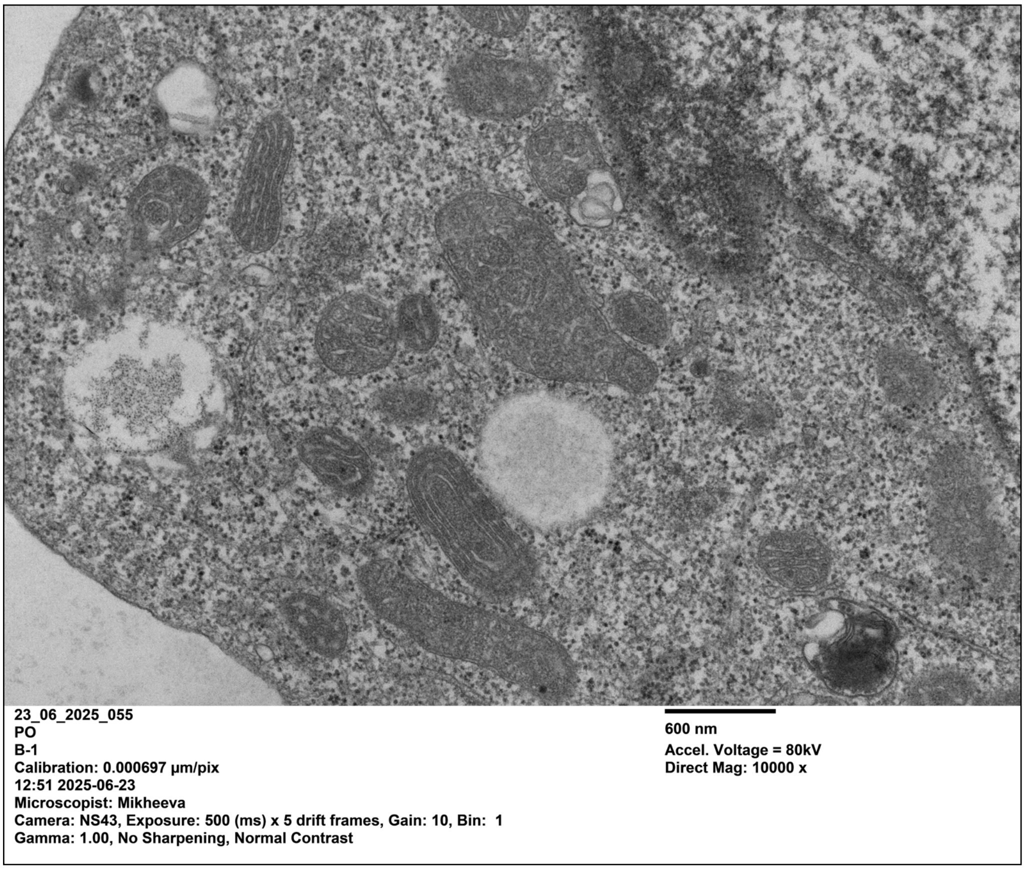

Figure S3. The original electron micrographs of Figure 3.

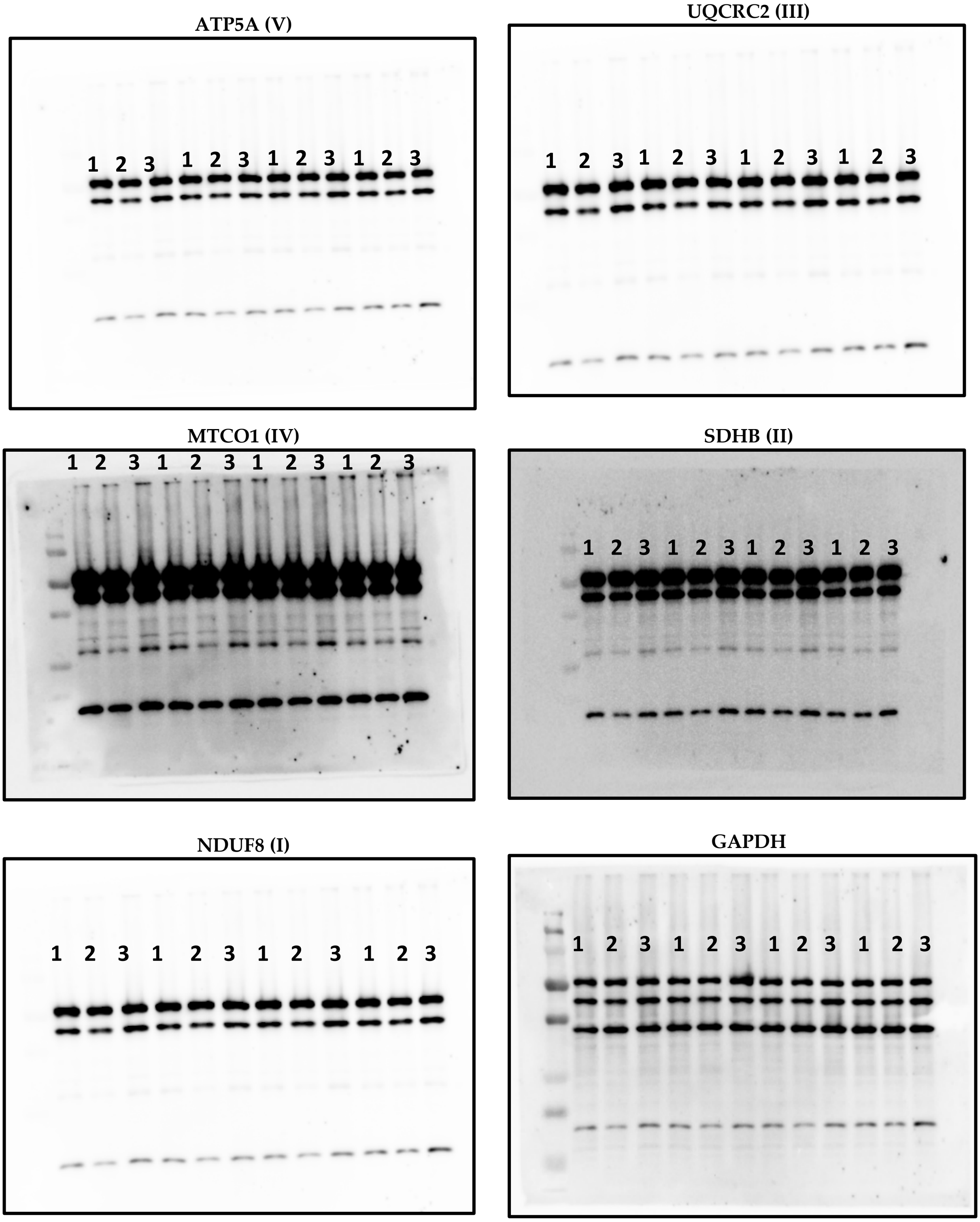

1 – THP-1LDC 2 – THP-1LPS 3 – THP-1HDC

Figure S4. The original WB images of Figure 4c.

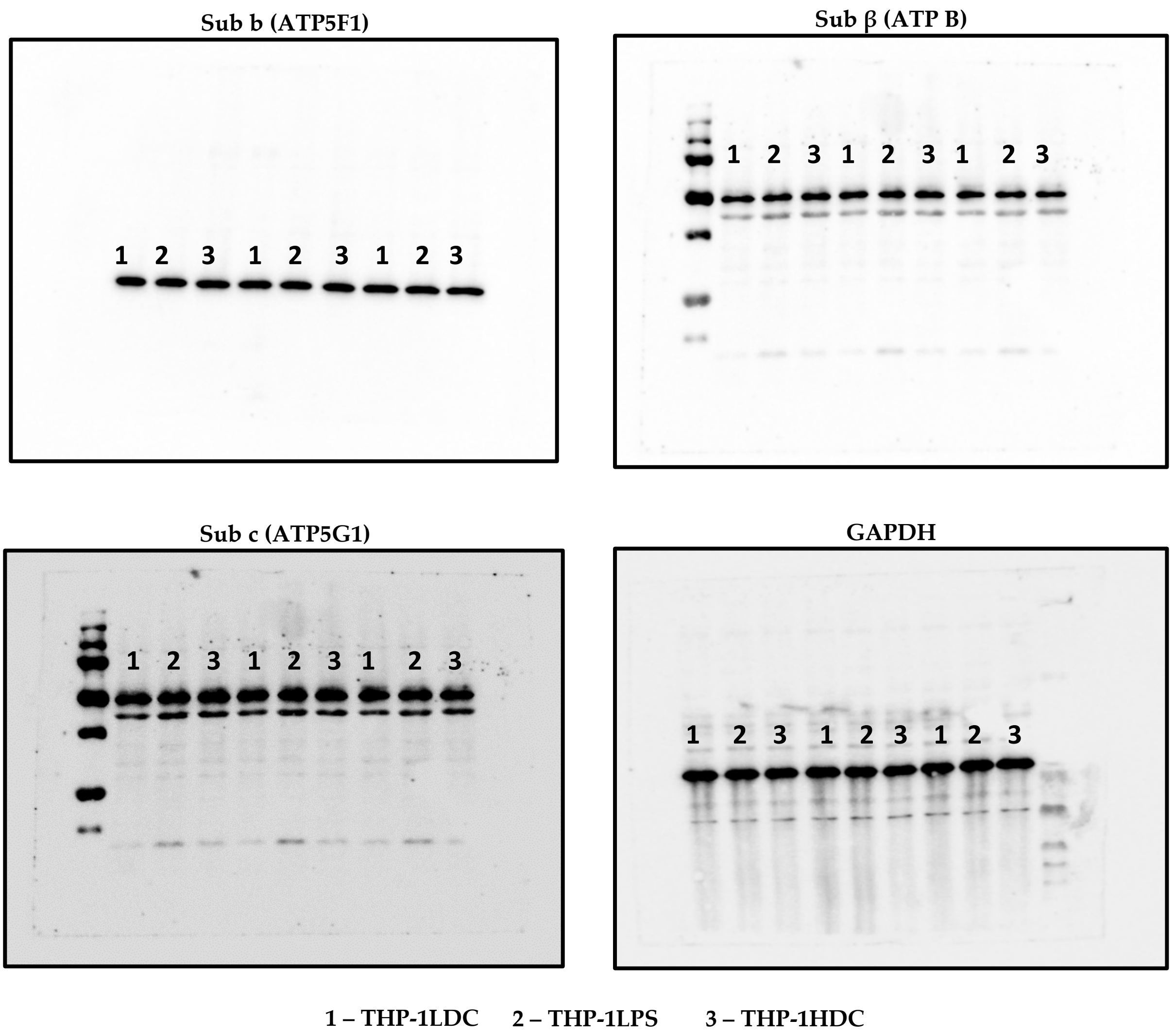

Figure S5. The original WB images of Figure 4e.

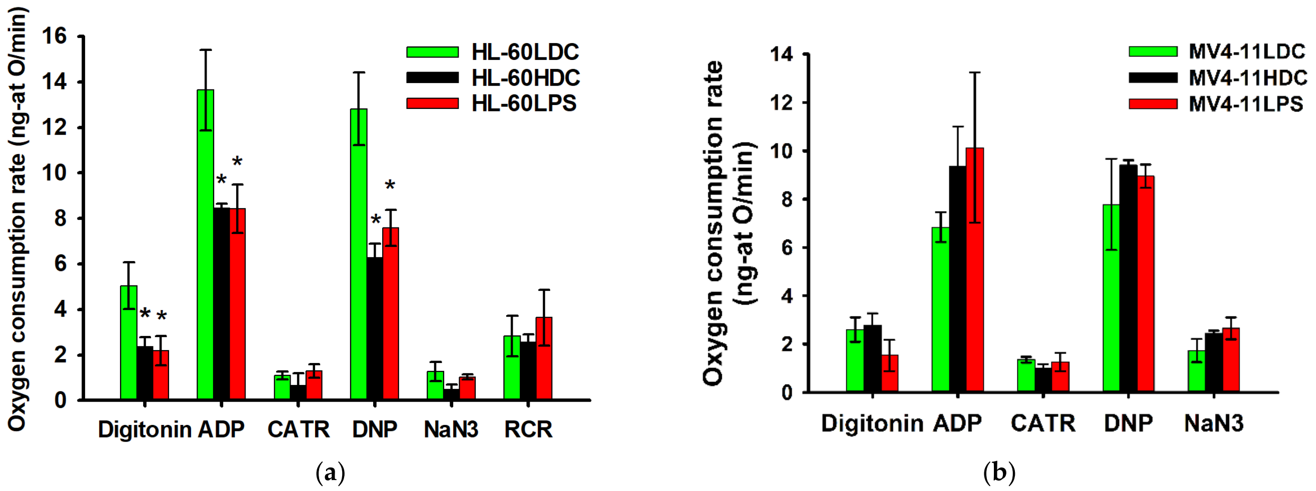

Figure S6. Mitochondrial respiratory chain activity in HL-60 (a) and MV4-11 (b) cells cultured under low-density conditions (LDC), in three-dimensional high-density cultures (HDC), or treated with LPS. The data is given as an average value ± SD. *—$p < 0.05$ in comparison with HL-60LDC or MV4-11LDC cells, respectively.

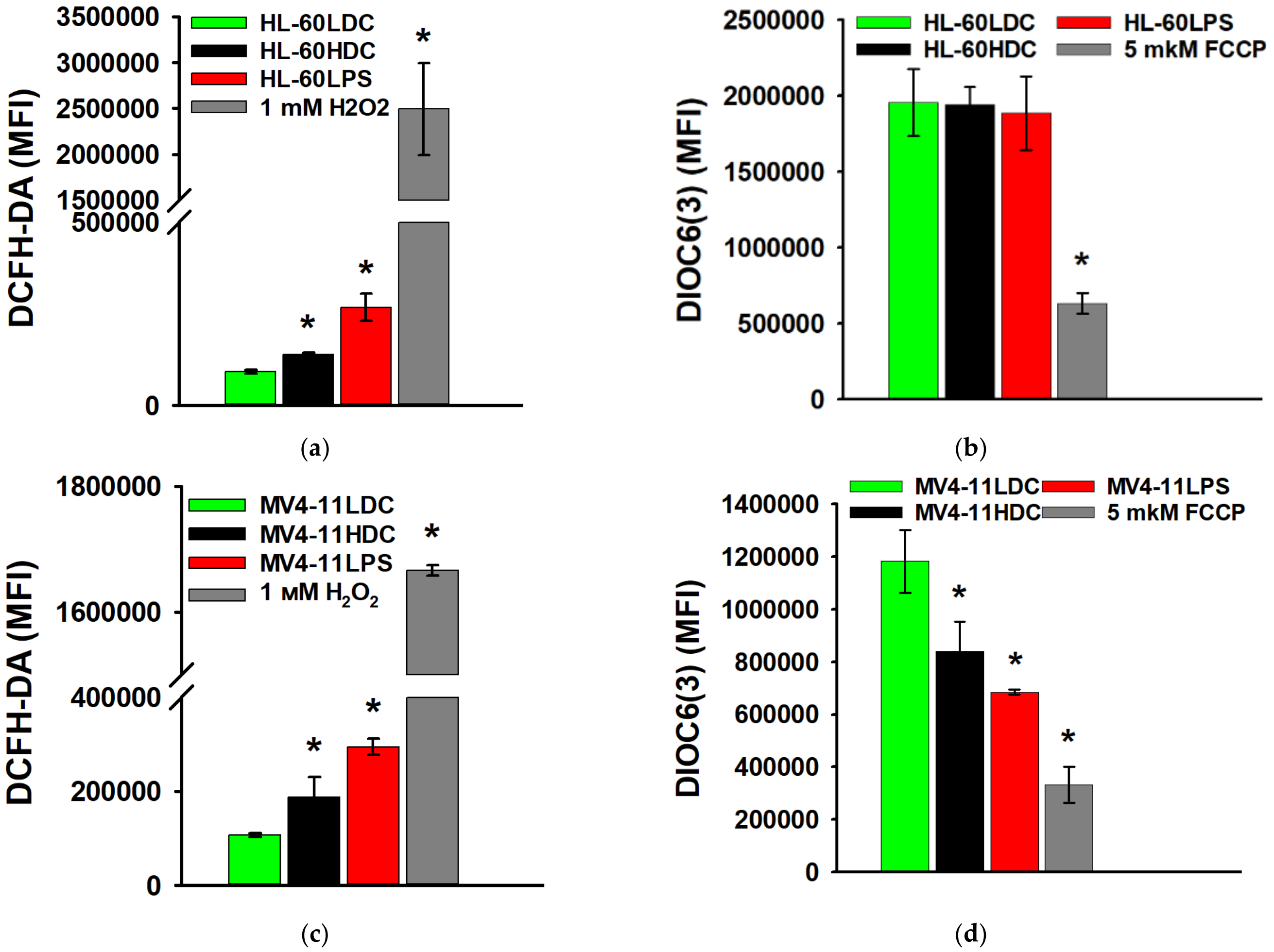

Figure S7. Intracellular ROS production (a, c) and mitochondrial potential (b, d) of HL-60 and MV4-11 cells cultured in low-density (LDC), three-dimensional high-density cultures (HDC) or after LPS (LPS) treatment. MFI is the mean fluorescence intensity of cells loaded with DCFH-DA or DIOC6(3). The data is given as an average value ± SD. *—p < 0.05 in comparison with HL-60LDC or MV4-11LDC cells, respectively.

Mfn2

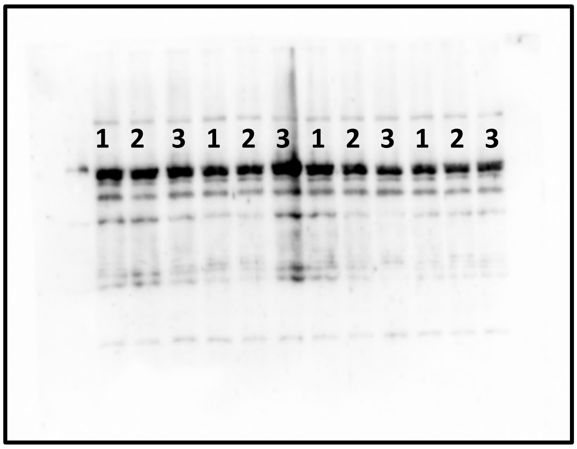

DRP1

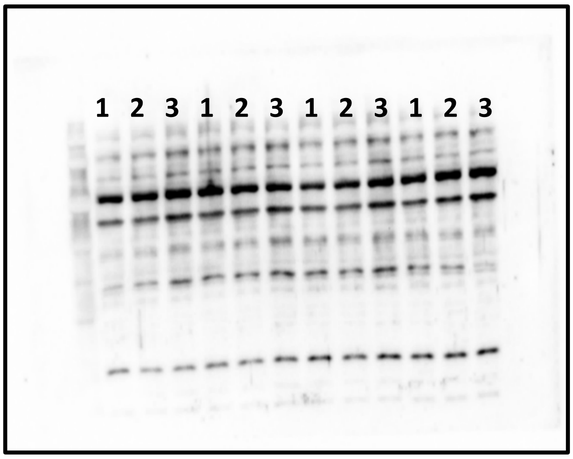

GAPDH

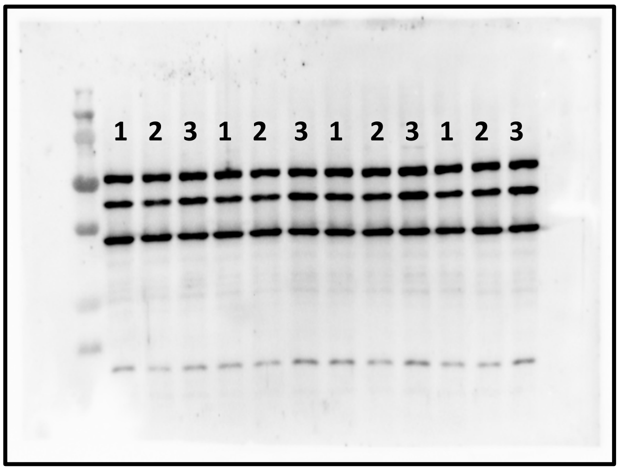

PINK

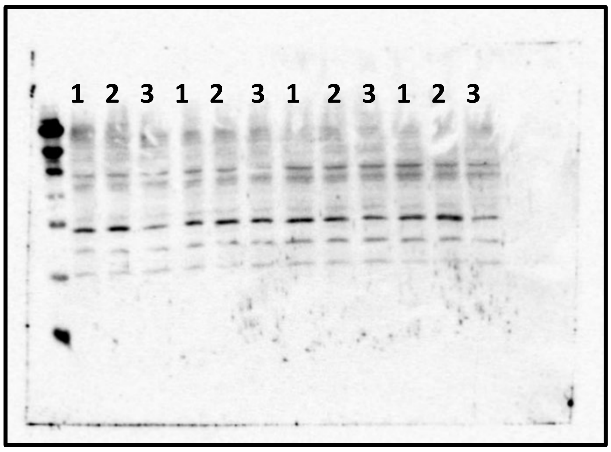

Parkin

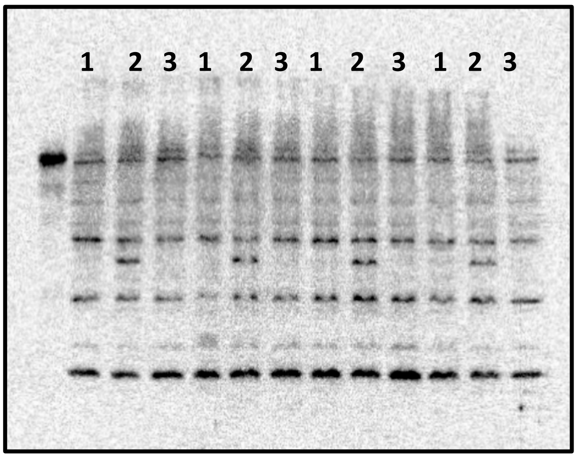

GAPDH

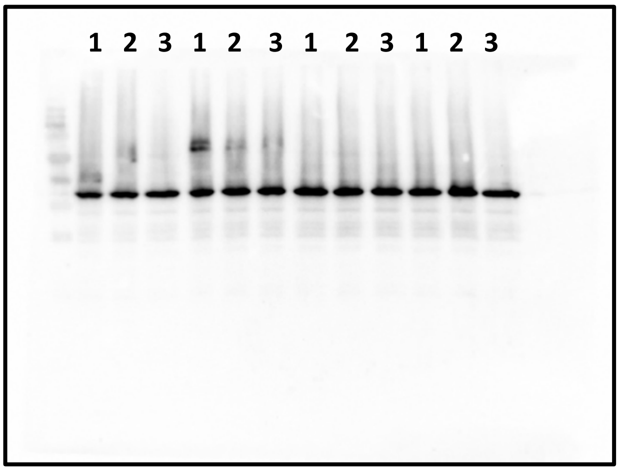

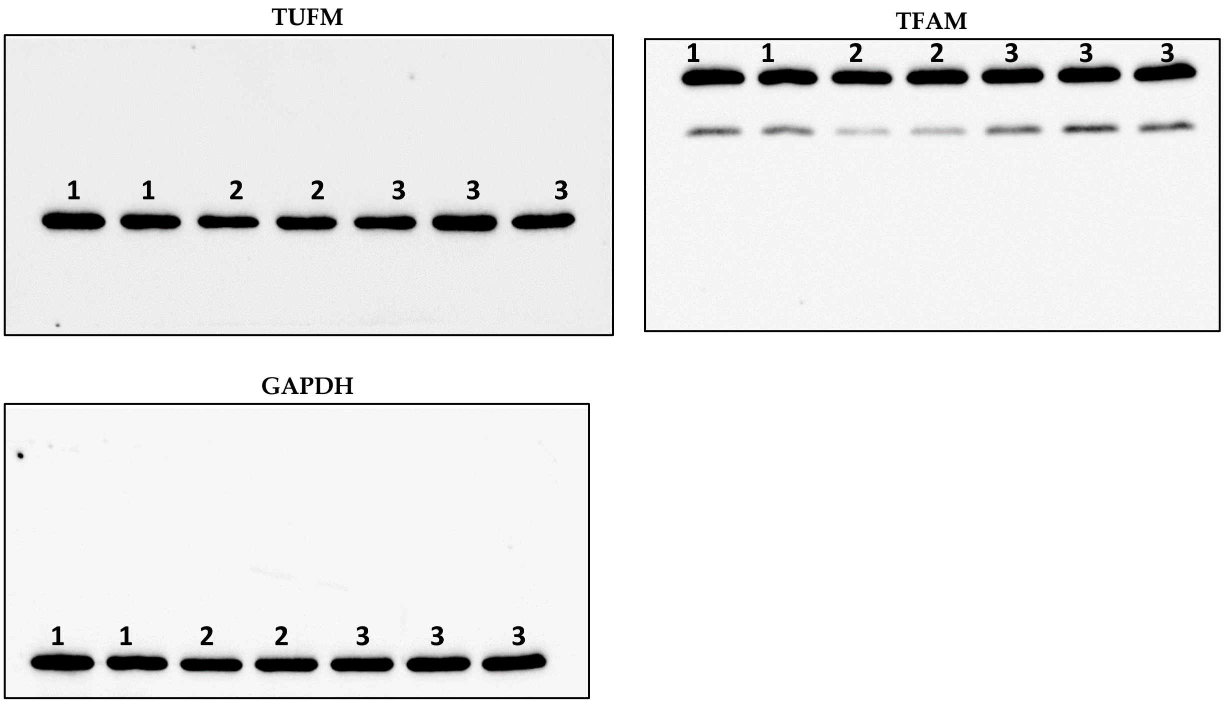

1 – THP-1LDC 2 – THP-1LPS 3 – THP-1HDC

Figure S8. The original WB images of Figure 6a.

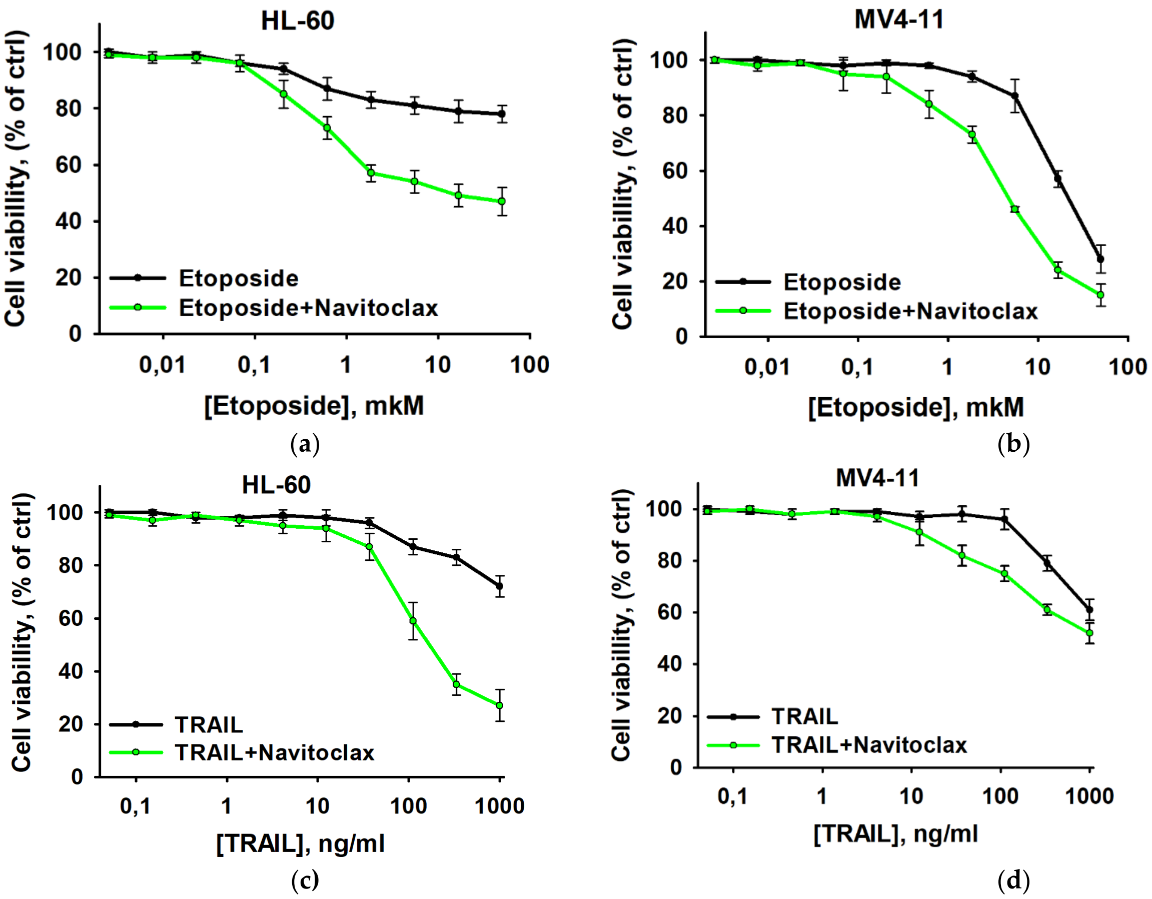

Figure S9. Navitoclax Reduces the Resistance of HL-60 and MV4-11 Cells in the HDC to Etoposide (a, c) and TRAIL (b, d). The data is given as an average value ± SD.

Table S1. Effect size matrices across multiple measured parameters

Intracellular ROS production

	THP-1LDC	THP-1LPS	THP-1HDC	H_2O_2
THP-1LDC	0	6,19	1,35	8,88
THP-1LPS	6,19	0	2,53	6,3
THP-1HDC	1,35	2,53	0	7,37
H_2O_2	8,88	6,3	7,37	0

	MV4-11LDC	MV4-11HDC	MV4-11LPS	H_2O_2
MV4-11LDC	0	1,43	11,83	191,35
MV4-11HDC	1,43	0	1,87	26,35
MV4-11LPS	11,83	1,87	0	79,52
H_2O_2	191,35	26,35	79,52	0

	HL-60LDC	HL-60HDC	HL-60LPS	H_2O_2
HL-60LDC	0	6,84	5,32	5,4
HL-60HDC	6,84	0	3,92	5,3
HL-60LPS	5,32	3,92	0	4,99
H_2O_2	5,4	5,3	4,99	0

Mitochondrial membrane potential

	THP-1LDC	THP-1LPS	THP-1HDC	FCCP
THP-1LDC	0	4,24	1,1	14,02
THP-1LPS	4,24	0	0,62	13,29
THP-1HDC	1,1	0,62	0	5,83
FCCP	14,02	13,29	5,83	0

	MV4-11LDC	MV4-11HDC	MV4-11LPS	FCCP
MV4-11LDC	0	2,64	4,67	6,93
MV4-11HDC	2,64	0	2,03	5,21
MV4-11LPS	4,67	2,03	0	5,68
FCCP	6,93	5,21	5,68	0

	HL-60LDC	HL-60HDC	HL-60LPS	FCCP
HL-60LDC	0	0,08	0,25	6,49
HL-60HDC	0,08	0	0,23	10,91
HL-60LPS	0,25	0,23	0	5,62
FCCP	6,49	10,91	5,62	0

**PINK**	THP-1LDC	THP-1HDC	THP-1LPS
THP-1LDC	0	1,77	2,17

THP-1HDC	1,77	0	4,68
THP-1LPS	2,17	4,68	0

**PARKIN**	THP-1LDC	THP-1HDC	THP-1LPS
THP-1LDC	0	2,22	2,72
THP-1HDC	2,22	0	0,52
THP-1LPS	2,72	0,52	0

**UQCRC2**	THP-1LDC	THP-1HDC	THP-1LPS
THP-1LDC	0	2,27	2,49
THP-1HDC	2,27	0	4,04
THP-1LPS	2,49	4,04	0

**MTCO1**	THP-1LDC	THP-1HDC	THP-1LPS
THP-1LDC	0	2,7	5,45
THP-1HDC	2,7	0	5,93
THP-1LPS	5,45	5,93	0

**SDHB**	THP-1LDC	THP-1HDC	THP-1LPS
THP-1LDC	0	2,31	2,24
THP-1HDC	2,31	0	3,28
THP-1LPS	2,24	3,28	0

**NDUF8**	THP-1LDC	THP-1HDC	THP-1LPS
THP-1LDC	0	0,75	3,62
THP-1HDC	0,75	0	4,82
THP-1LPS	3,62	4,82	0

**ATP5G1**	THP-1LDC	THP-1HDC	THP-1LPS
THP-1LDC	0	2,15	5,62
THP-1HDC	2,15	0	3,61
THP-1LPS	5,62	3,61	0

**DRP1**	THP-1LDC	THP-1HDC	THP-1LPS
THP-1LDC	0	2,67	0,76
THP-1HDC	2,67	0	1,5
THP-1LPS	0,76	1,5	0

**Lactate concentration**	THP-1LDC	THP-1LPS	THP-1HDC
THP-1LDC	0	0,6	0,63
THP-1LPS	0,6	0	0,37
THP-1HDC	0,63	0,37	0

Mitochondrial respiratory chain activity

**THP-1 (digitonin)**	THP-1LDC	THP-1HDC	THP-1LPS
THP-1LDC	0	0,62	1,54
THP-1HDC	0,62	0	0,13
THP-1LPS	1,54	0,13	0

**THP-1 (ADP)**	THP-1LDC	THP-1HDC	THP-1LPS
THP-1LDC	0	6,14	5,41
THP-1HDC	6,14	0	2,42
THP-1LPS	5,41	2,42	0

**THP-1 (DNP)**	THP-1LDC	THP-1HDC	THP-1LPS
THP-1LDC	0	3,37	3,98
THP-1HDC	3,37	0	0,35
THP-1LPS	3,98	0,35	0

**HL-60 (digitonin)**	HL-60LDC	HL-60HDC	HL-60LPS
HL-60LDC	0	1,71	2,16
HL-60HDC	1,71	0	1,02
HL-60LPS	2,16	1,02	0

**HL-60 (ADP)**	HL-60LDC	HL-60HDC	HL-60LPS
HL-60LDC	0	3,3	2,86
HL-60HDC	3,3	0	0,02
HL-60LPS	2,86	0,02	0

**HL-60 (DNP)**	HL-60LDC	HL-60HDC	HL-60LPS
HL-60LDC	0	3,58	3,31
HL-60HDC	3,58	0	0,33
HL-60LPS	3,31	0,33	0

Table S2. MMRGs with differential expression in THP-1HDC AML cells compared to THP-1LDC cells (FDR < 0.05).

**gene_id**	**name**	**description**	**log2FoldChange**	**pvalue**	**padj**
ENSG00000104765	BNIP3L	BCL2 interacting protein 3 like	1,058338052	6,44E-12	2,64E-10
ENSG00000167107	ACSF2	acyl-CoA synthetase family member 2	0,992868833	6,41E-08	0,00000115
ENSG00000163644	PPM1K	protein phosphatase%2C Mg2+/Mn2+ dependent 1K	0,989778856	1,61E-08	0,000000331
ENSG00000183044	ABAT	4-aminobutyrate aminotransferase	0,918714138	0,000303267	0,002123068
ENSG00000110011	DNAJC4	DnaJ heat shock protein family (Hsp40) member C4	0,860018405	8,47E-08	0,00000147

ENSG00000169100	SLC25A6	solute carrier family 25 member 6	0,789299785	2,99E-28	1,27E-25
ENSG00000100596	SPTLC2	serine palmitoyltransferase long chain base subunit 2	0,785564313	2,6E-11	9,6E-10
ENSG00000183773	AIFM3	apoptosis inducing factor%2C mitochondria associated 3	0,767545919	0,000649513	0,004098222
ENSG00000135241	PNPLA8	patatin like phospholipase domain containing 8	0,747541901	0,0000392	0,000361073
ENSG00000050426	LETMD1	LETM1 domain containing 1	0,706225056	5,41E-10	1,54E-08
ENSG00000121310	ECHDC2	enoyl-CoA hydratase domain containing 2	0,659070089	1,24E-10	3,97E-09
ENSG00000143224	PPOX	protoporphyrinogen oxidase	0,638097683	0,003615157	0,017545242
ENSG00000155368	DBI	diazepam binding inhibitor%2C acyl-CoA binding protein	0,632406163	2,46E-13	1,36E-11
ENSG00000122971	ACADS	acyl-CoA dehydrogenase short chain	0,565482233	0,000169418	0,001277135
ENSG00000181192	DHTKD1	dehydrogenase E1 and transketolase domain containing 1	0,56543006	0,00000012	0,000002
ENSG00000119711	ALDH6A1	aldehyde dehydrogenase 6 family member A1	0,56066292	0,000485827	0,003181322
ENSG00000163864	NMNAT3	nicotinamide nucleotide adenylyltransferase 3	0,559862652	0,000270947	0,001924557
ENSG00000110455	ACCS	1-aminocyclopropane-1-carboxylate synthase homolog (inactive)	0,522399797	0,000075	0,000630433
ENSG00000023330	ALAS1	5'-aminolevulinate synthase 1	0,503489827	0,00655079	0,028550166
ENSG00000166816	LDHD	lactate dehydrogenase D	0,48805242	0,007635746	0,032378121
ENSG00000124098	FAM210B	family with sequence similarity 210 member B	0,483086841	0,011520613	0,044902075
ENSG00000174606	ANGEL2	angel homolog 2	0,479441405	0,000882308	0,005327037
ENSG00000181035	SLC25A42	solute carrier family 25 member 42	0,463062751	0,010603967	0,042025831
ENSG00000101986	ABCD1	ATP binding cassette subfamily D member 1	0,451009495	0,001730853	0,009516076
ENSG00000107819	SFXN3	sideroflexin 3	0,394454237	0,00000333	0,0000398
ENSG00000100577	GSTZ1	glutathione S-transferase zeta 1	0,384841081	0,012160814	0,046952371
ENSG00000088888	MAVS	mitochondrial antiviral signaling protein	0,374527116	0,000000776	0,0000108
ENSG00000167996	FTH1	ferritin heavy chain 1	0,372497449	0,000545114	0,003522566
ENSG00000095321	CRAT	carnitine O-acetyltransferase	0,349306966	0,00706512	0,030379323
ENSG00000081791	KIAA0141	KIAA0141	0,321147011	0,002918567	0,014729365
ENSG00000171791	BCL2	BCL2%2C apoptosis regulator	0,320244093	0,000000411	0,0000061

ENSG00000084092	NOA1	nitric oxide associated 1	0,318445111	0,003959781	0,018904114
ENSG00000067064	IDI1	isopentenyl-diphosphate delta isomerase 1	0,303954669	0,006984725	0,030102471
ENSG00000115204	MPV17	MPV17%2C mitochondrial inner membrane protein	0,301746893	0,010500179	0,041708106
ENSG00000104325	DECR1	2%2C4-dienoyl-CoA reductase 1	0,266981569	0,000656065	0,004133617
ENSG00000147853	AK3	adenylate kinase 3	0,266524585	0,011605472	0,04515295
ENSG00000105701	FKBP8	FK506 binding protein 8	0,263769241	0,001437527	0,008103097
ENSG00000164830	OXR1	oxidation resistance 1	0,257800759	0,009732481	0,039185965
ENSG00000105819	PMPCB	peptidase%2C mitochondrial processing beta subunit	0,256063646	0,011809763	0,045839017
ENSG00000093010	COMT	catechol-O-methyltransferase	0,232871281	0,002315882	0,012105498
ENSG00000188554	NBR1	NBR1%2C autophagy cargo receptor	0,217528227	0,012269889	0,047327962
ENSG00000155463	OXA1L	OXA1L%2C mitochondrial inner membrane protein	0,215432779	0,0031146	0,015504676
ENSG00000198786	MT-ND5	mitochondrially encoded NADH:ubiquinone oxidoreductase core subunit 5	-0,147799382	0,003974789	0,018957854
ENSG00000073578	SDHA	succinate dehydrogenase complex flavoprotein subunit A	-0,170349982	0,008639321	0,035654999
ENSG00000075415	SLC25A3	solute carrier family 25 member 3	-0,17311592	0,004038639	0,01920396
ENSG00000182199	SHMT2	serine hydroxymethyltransferase 2	-0,179289793	0,008641502	0,035654999
ENSG00000198840	MT-ND3	mitochondrially encoded NADH:ubiquinone oxidoreductase core subunit 3	-0,183248002	0,006240141	0,027432442
ENSG00000169710	FASN	fatty acid synthase	-0,184718841	0,005138926	0,023510094
ENSG00000165672	PRDX3	peroxiredoxin 3	-0,184863594	0,003222022	0,015907071
ENSG00000146701	MDH2	malate dehydrogenase 2	-0,19714435	0,005170258	0,023623826
ENSG00000198888	MT-ND1	mitochondrially encoded NADH:ubiquinone oxidoreductase core subunit 1	-0,198475072	0,003179268	0,015757022
ENSG00000138095	LRPPRC	leucine rich pentatricopeptide repeat containing	-0,208095197	0,000603425	0,003858565
ENSG00000106153	CHCHD2	coiled-coil-helix-coiled-coil-helix domain containing 2	-0,216114039	0,009553803	0,038631998
ENSG00000130414	NDUFA10	NADH:ubiquinone oxidoreductase subunit A10	-0,216889497	0,010727473	0,042410881
ENSG00000154723	ATP5J	ATP synthase%2C H+ transporting%2C mitochondrial Fo complex subunit F6	-0,220779113	0,008984596	0,036736642
ENSG00000121691	CAT	catalase	-0,221838248	0,000168869	0,00127373

ENSG00000141385	AFG3L2	AFG3 like matrix AAA peptidase subunit 2	-0,225211222	0,005470829	0,024713185
ENSG00000121057	AKAP1	A-kinase anchoring protein 1	-0,233248987	0,007403203	0,031575164
ENSG00000178741	COX5A	cytochrome c oxidase subunit 5A	-0,234388535	0,000887891	0,005353379
ENSG00000143774	GUK1	guanylate kinase 1	-0,236894139	0,005932734	0,026318386
ENSG00000004455	AK2	adenylate kinase 2	-0,242687478	0,000549083	0,003546136
ENSG00000147471	PLPBP	pyridoxal phosphate binding protein	-0,242831877	0,005362716	0,024330945
ENSG00000152234	ATP5A1	ATP synthase%2C H+ transporting%2C mitochondrial F1 complex%2C alpha subunit 1%2C cardiac muscle	-0,24572717	0,0000027	0,0000329
ENSG00000274523	RCC1L	RCC1 like	-0,245841355	0,004540555	0,021199995
ENSG00000115159	GPD2	glycerol-3-phosphate dehydrogenase 2	-0,246921661	0,003355356	0,016460381
ENSG00000075239	ACAT1	acetyl-CoA acetyltransferase 1	-0,251656456	0,00520513	0,023753921
ENSG00000169738	DCXR	dicarbonyl and L-xylulose reductase	-0,257923823	0,013118163	0,049856504
ENSG00000178057	NDUFAF3	NADH:ubiquinone oxidoreductase complex assembly factor 3	-0,263167598	0,002932667	0,014772202
ENSG00000156709	AIFM1	apoptosis inducing factor mitochondria associated 1	-0,265939683	0,003179657	0,015757022
ENSG00000100335	MIEF1	mitochondrial elongation factor 1	-0,26599769	0,000158005	0,001205864
ENSG00000215021	PHB2	prohibitin 2	-0,2669482	0,000909206	0,005456894
ENSG00000173085	COQ2	coenzyme Q2%2C polyprenyltransferase	-0,267663887	0,012823529	0,048979482
ENSG00000111716	LDHB	lactate dehydrogenase B	-0,272585902	0,0000207	0,00020441
ENSG00000062485	CS	citrate synthase	-0,27307385	0,000231671	0,001672655
ENSG00000110074	FOXRED1	FAD dependent oxidoreductase domain containing 1	-0,273906688	0,006385931	0,027961284
ENSG00000100823	APEX1	apurinic/apyrimidinic endodeoxyribonuclease 1	-0,279064774	0,0000352	0,00032719
ENSG00000082212	ME2	malic enzyme 2	-0,286727456	0,0000386	0,000356669
ENSG00000198763	MT-ND2	mitochondrially encoded NADH:ubiquinone oxidoreductase core subunit 2	-0,289355591	0,0000125	0,000130897
ENSG00000123472	ATPAF1	ATP synthase mitochondrial F1 complex assembly factor 1	-0,292382785	0,005170965	0,023623826
ENSG00000148175	STOM	stomatin	-0,292924256	0,000290083	0,002041753
ENSG00000140521	POLG	DNA polymerase gamma%2C catalytic subunit	-0,294017688	0,000342639	0,002351309

ENSG00000101166	PRELID3B	PRELI domain containing 3B	-0,294052827	0,000500054	0,003271228
ENSG00000173141	MRPL57	mitochondrial ribosomal protein L57	-0,298558275	0,012525876	0,048124231
ENSG00000167468	GPX4	glutathione peroxidase 4	-0,299551516	0,00029596	0,002078671
ENSG00000051341	POLQ	DNA polymerase theta	-0,305251542	0,002854955	0,014447104
ENSG00000069998	HDHD5	haloacid dehalogenase like hydrolase domain containing 5	-0,306099204	0,004601951	0,021441018
ENSG00000114021	NIT2	nitrilase family member 2	-0,307678765	0,002088409	0,011101633
ENSG00000177150	FAM210A	family with sequence similarity 210 member A	-0,310434308	0,010501494	0,041708106
ENSG00000182180	MRPS16	mitochondrial ribosomal protein S16	-0,312133948	0,000308873	0,002154417
ENSG00000103202	NME4	NME/NM23 nucleoside diphosphate kinase 4	-0,313081908	0,0000287	0,000273588
ENSG00000107959	PITRM1	pitrilysin metallopeptidase 1	-0,313158306	0,000523216	0,003400774
ENSG00000107951	MTPAP	mitochondrial poly(A) polymerase	-0,313774889	0,010181153	0,040644323
ENSG00000143374	TARS2	threonyl-tRNA synthetase 2%2C mitochondrial (putative)	-0,314006901	0,003990274	0,0190152
ENSG00000067704	IARS2	isoleucyl-tRNA synthetase 2%2C mitochondrial	-0,314487	0,00000343	0,0000409
ENSG00000091483	FH	fumarate hydratase	-0,314852613	0,0000467	0,000418946
ENSG00000247626	MARS2	methionyl-tRNA synthetase 2%2C mitochondrial	-0,315861482	0,012965472	0,049435555
ENSG00000116459	ATP5F1	ATP synthase%2C H+ transporting%2C mitochondrial Fo complex subunit B1	-0,317408298	0,000000456	0,00000668
ENSG00000184428	TOP1MT	DNA topoisomerase I mitochondrial	-0,322347382	0,000257329	0,00183874
ENSG00000164347	GFM2	G elongation factor mitochondrial 2	-0,32234785	0,003789878	0,01826524
ENSG00000116288	PARK7	Parkinsonism associated deglycase	-0,326411059	0,0000733	0,000618004
ENSG00000112996	MRPS30	mitochondrial ribosomal protein S30	-0,326688535	0,0000109	0,00011546
ENSG00000119421	NDUFA8	NADH:ubiquinone oxidoreductase subunit A8	-0,327320897	0,005582923	0,025096457
ENSG00000099624	ATP5D	ATP synthase%2C H+ transporting%2C mitochondrial F1 complex%2C delta subunit	-0,329476429	0,000122454	0,000965691
ENSG00000002549	LAP3	leucine aminopeptidase 3	-0,33167795	0,000144694	0,001117645
ENSG00000005156	LIG3	DNA ligase 3	-0,332598943	0,005322431	0,024189861
ENSG00000132463	GRSF1	G-rich RNA sequence binding factor 1	-0,335049471	0,00000421	0,0000491

ENSG00000084090	STARD7	StAR related lipid transfer domain containing 7	-0,335498574	0,000000765	0,0000107
ENSG00000110717	NDUFS8	NADH:ubiquinone oxidoreductase core subunit S8	-0,335598757	0,000629385	0,003986506
ENSG00000168393	DTYMK	deoxythymidylate kinase	-0,337151487	0,001294122	0,007408977
ENSG00000074071	MRPS34	mitochondrial ribosomal protein S34	-0,339133156	0,000190602	0,001409386
ENSG00000136522	MRPL47	mitochondrial ribosomal protein L47	-0,339693526	0,004115349	0,019540486
ENSG00000112110	MRPL18	mitochondrial ribosomal protein L18	-0,342768401	0,000147889	0,001136982
ENSG00000168653	NDUFS5	NADH:ubiquinone oxidoreductase subunit S5	-0,344540774	0,001283191	0,007352793
ENSG00000117528	ABCD3	ATP binding cassette subfamily D member 3	-0,344660052	0,004305763	0,020334216
ENSG00000164405	UQCRQ	ubiquinol-cytochrome c reductase complex III subunit VII	-0,344821645	0,000883134	0,005329579
ENSG00000196236	XPNPEP3	X-prolyl aminopeptidase 3	-0,345578329	0,002961194	0,014867067
ENSG00000124608	AARS2	alanyl-tRNA synthetase 2%2C mitochondrial	-0,347149214	0,004437554	0,020815071
ENSG00000063241	ISOC2	isochorismatase domain containing 2	-0,347236951	0,002057859	0,010963973
ENSG00000140990	NDUFB10	NADH:ubiquinone oxidoreductase subunit B10	-0,348334528	0,000113939	0,000907788
ENSG00000085760	MTIF2	mitochondrial translational initiation factor 2	-0,348368828	0,000667885	0,004192037
ENSG00000178074	C2orf69	chromosome 2 open reading frame 69	-0,348778372	0,007719833	0,032629559
ENSG00000165678	GHITM	growth hormone inducible transmembrane protein	-0,34949462	0,000000324	0,00000493
ENSG00000061794	MRPS35	mitochondrial ribosomal protein S35	-0,35056537	0,005439466	0,024602836
ENSG00000130348	QRSL1	glutaminyl-tRNA synthase (glutamine-hydrolyzing)-like 1	-0,351047896	0,000789725	0,004836837
ENSG00000215012	RTL10	retrotransposon Gag like 10	-0,352098683	0,003835416	0,01843072
ENSG00000083720	OXCT1	3-oxoacid CoA-transferase 1	-0,353637738	0,0000064	0,0000713
ENSG00000102967	DHODH	dihydroorotate dehydrogenase (quinone)	-0,354982156	0,012296057	0,0473616
ENSG00000266472	MRPS21	mitochondrial ribosomal protein S21	-0,355696274	0,000846554	0,005148957
ENSG00000136270	TBRG4	transforming growth factor beta regulator 4	-0,357510116	0,0000167	0,000169039
ENSG00000115364	MRPL19	mitochondrial ribosomal protein L19	-0,358305373	0,001074162	0,006322954
ENSG00000119912	IDE	insulin degrading enzyme	-0,361409381	0,0000241	0,000234333
ENSG00000132153	DHX30	DExH-box helicase 30	-0,362233498	0,00000234	0,000029

ENSG00000164896	FASTK	Fas activated serine/threonine kinase	-0,362904796	0,000681072	0,004255663
ENSG00000131368	MRPS25	mitochondrial ribosomal protein S25	-0,364224542	0,000179364	0,001335289
ENSG00000065154	OAT	ornithine aminotransferase	-0,366170432	0,00000742	0,0000816
ENSG00000171202	TMEM126A	transmembrane protein 126A	-0,367518873	0,012639338	0,048430558
ENSG00000037474	NSUN2	NOP2/Sun RNA methyltransferase family member 2	-0,368411712	2,16E-09	5,42E-08
ENSG00000134905	CARS2	cysteinyl-tRNA synthetase 2%2C mitochondrial	-0,370043186	0,000658486	0,004144905
ENSG00000174917	C19orf70	chromosome 19 open reading frame 70	-0,375971009	0,006399853	0,028003613
ENSG00000198431	TXNRD1	thioredoxin reductase 1	-0,376046627	4,46E-08	0,000000832
ENSG00000148334	PTGES2	prostaglandin E synthase 2	-0,376962202	0,000534173	0,003463443
ENSG00000169020	ATP5I	ATP synthase%2C H+ transporting%2C mitochondrial Fo complex subunit E	-0,37851696	0,006548623	0,028550166
ENSG00000126814	TRMT5	tRNA methyltransferase 5	-0,378935507	0,003208957	0,015854464
ENSG00000100412	ACO2	aconitase 2	-0,378990164	0,000000644	0,00000921
ENSG00000135297	MTO1	mitochondrial tRNA translation optimization 1	-0,384484773	0,0000312	0,00029536
ENSG00000151014	NOCT	nocturnin	-0,385399184	0,000717574	0,004451575
ENSG00000100294	MCAT	malonyl-CoA-acyl carrier protein transacylase	-0,385933087	0,009054422	0,03695327
ENSG00000160688	FLAD1	flavin adenine dinucleotide synthetase 1	-0,388758123	0,00000994	0,000106129
ENSG00000184076	UQCR10	ubiquinol-cytochrome c reductase%2C complex III subunit X	-0,389713508	0,000116929	0,000928798
ENSG00000167792	NDUFV1	NADH:ubiquinone oxidoreductase core subunit V1	-0,390864788	0,00000025	0,00000389
ENSG00000153574	RPIA	ribose 5-phosphate isomerase A	-0,391649867	0,000622857	0,003958496
ENSG00000065427	KARS	lysyl-tRNA synthetase	-0,392388302	0,000000026	0,00000051
ENSG00000154174	TOMM70	translocase of outer mitochondrial membrane 70	-0,392926682	8,48E-08	0,00000147
ENSG00000177542	SLC25A22	solute carrier family 25 member 22	-0,393795478	0,000317461	0,002203807
ENSG00000213585	VDAC1	voltage dependent anion channel 1	-0,393950353	5,14E-09	0,000000117
ENSG00000162129	CLPB	ClpB homolog%2C mitochondrial AAA ATPase chaperonin	-0,396234496	0,0000184	0,000184212
ENSG00000183605	SFXN4	sideroflexin 4	-0,397086689	0,001025144	0,006065073
ENSG00000111275	ALDH2	aldehyde dehydrogenase 2 family (mitochondrial)	-0,397560979	0,001394175	0,007891363

ENSG00000101084	C20orf24	chromosome 20 open reading frame 24	-0,397769968	0,000832397	0,005081634
ENSG00000167112	TRUB2	TruB pseudouridine synthase family member 2	-0,397843896	0,00029752	0,002087395
ENSG00000154518	ATP5G3	ATP synthase%2C H+ transporting%2C mitochondrial Fo complex subunit C3 (subunit 9)	-0,39801962	2,98E-09	7,23E-08
ENSG00000168924	LETM1	leucine zipper and EF-hand containing transmembrane protein 1	-0,398703947	7,13E-08	0,00000127
ENSG00000196365	LONP1	lon peptidase 1%2C mitochondrial	-0,402776866	3,42E-08	0,000000657
ENSG00000106105	GARS	glycyl-tRNA synthetase	-0,40313815	2,45E-10	7,48E-09
ENSG00000105552	BCAT2	branched chain amino acid transaminase 2	-0,403457156	0,003083097	0,015376933
ENSG00000082515	MRPL22	mitochondrial ribosomal protein L22	-0,404184523	0,001377404	0,007814396
ENSG00000182768	NGRN	neugrin%2C neurite outgrowth associated	-0,404549002	0,002268646	0,011896233
ENSG00000154719	MRPL39	mitochondrial ribosomal protein L39	-0,405684336	0,002818787	0,014291553
ENSG00000097021	ACOT7	acyl-CoA thioesterase 7	-0,405703329	0,002466321	0,012780203
ENSG00000142168	SOD1	superoxide dismutase 1	-0,406710689	1,42E-09	3,65E-08
ENSG00000145494	NDUFS6	NADH:ubiquinone oxidoreductase subunit S6	-0,40758089	1,55E-09	3,94E-08
ENSG00000168827	GFM1	G elongation factor mitochondrial 1	-0,408725588	0,00000342	0,0000409
ENSG00000106554	CHCHD3	coiled-coil-helix-coiled-coil-helix domain containing 3	-0,408778703	0,000000169	0,00000274
ENSG00000108064	TFAM	transcription factor A%2C mitochondrial	-0,41277968	0,0000186	0,000186041
ENSG00000078668	VDAC3	voltage dependent anion channel 3	-0,412820158	4,1E-09	9,62E-08
ENSG00000243927	MRPS6	mitochondrial ribosomal protein S6	-0,415119362	0,009975083	0,040015861
ENSG00000165637	VDAC2	voltage dependent anion channel 2	-0,415796107	0,000492627	0,003224247
ENSG00000100347	SAMM50	SAMM50 sorting and assembly machinery component	-0,415986486	0,00000297	0,0000358
ENSG00000101181	MTG2	mitochondrial ribosome associated GTPase 2	-0,41667844	0,001199914	0,006929909
ENSG00000174547	MRPL11	mitochondrial ribosomal protein L11	-0,416876759	0,000669326	0,004198746
ENSG00000159445	THEM4	thioesterase superfamily member 4	-0,417616091	0,006962145	0,030014987
ENSG00000138078	PREPL	prolyl endopeptidase-like	-0,423021219	0,000188939	0,001398662
ENSG00000165060	FXN	frataxin	-0,424135571	0,005089298	0,023327353

ENSG0000006 3176	SPHK2	sphingosine kinase 2	-0,428233819	0,005304 787	0,024118 001
ENSG0000000 4864	SLC25A13	solute carrier family 25 member 13	-0,430807236	0,002986 415	0,014971 509
ENSG0000016 5264	NDUFB6	NADH:ubiquinone oxidoreductase subunit B6	-0,431299439	0,002030 42	0,010845 935
ENSG0000010 8439	PNPO	pyridoxamine 5'-phosphate oxidase	-0,431560464	0,001152 675	0,006690 002
ENSG0000006 0762	MPC1	mitochondrial pyruvate carrier 1	-0,431640541	0,000060 1	0,000520 92
ENSG0000008 8766	CRLS1	cardiolipin synthase 1	-0,434298369	0,002081 555	0,011074 139
ENSG0000000 7923	DNAJC11	DnaJ heat shock protein family (Hsp40) member C11	-0,435644805	0,000196 045	0,001445 57
ENSG0000012 5901	MRPS26	mitochondrial ribosomal protein S26	-0,437258549	0,005925 333	0,026294 415
ENSG0000009 1140	DLD	dihydrolipoamide dehydrogenase	-0,437475392	1,93E-08	0,000000 391
ENSG0000013 1828	PDHA1	pyruvate dehydrogenase E1 alpha 1 subunit	-0,437751498	0,000002 09	0,000026 1
ENSG0000013 8382	METTL5	methyltransferase like 5	-0,438548707	0,000272 83	0,001936 891
ENSG0000011 1775	COX6A1	cytochrome c oxidase subunit 6A1	-0,43926142	0,001656 12	0,009162 612
ENSG0000018 4752	NDUFA12	NADH:ubiquinone oxidoreductase subunit A12	-0,44135495	0,000141 016	0,001089 872
ENSG0000000 6744	ELAC2	elaC ribonuclease Z 2	-0,441672223	0,000005 36	0,000060 6
ENSG0000010 7815	TWNK	twinkle mtDNA helicase	-0,443264909	0,000109 356	0,000877 114
ENSG0000013 7288	UQCC2	ubiquinol-cytochrome c reductase complex assembly factor 2	-0,44384751	0,000254 429	0,001819 995
ENSG0000010 8528	SLC25A11	solute carrier family 25 member 11	-0,444289411	0,000047 3	0,000423 696
ENSG0000016 7699	GLOD4	glyoxalase domain containing 4	-0,444594881	0,000163 048	0,001237 626
ENSG0000010 8826	MRPL27	mitochondrial ribosomal protein L27	-0,446631965	0,001663 217	0,009194 148
ENSG0000016 4466	SFXN1	sideroflexin 1	-0,447221716	7,75E-09	0,000000 171
ENSG0000013 7767	SQOR	sulfide quinone oxidoreductase	-0,453045852	0,000181 257	0,001347 092
ENSG0000025 4858	MPV17L2	MPV17 mitochondrial inner membrane protein like 2	-0,453888892	0,009352 081	0,037944 541
ENSG0000018 2810	DDX28	DEAD-box helicase 28	-0,454637159	0,001863 531	0,010118 728
ENSG0000013 5972	MRPS9	mitochondrial ribosomal protein S9	-0,455014979	0,001028 942	0,006081 243
ENSG0000017 1865	RNASEH1	ribonuclease H1	-0,455892463	0,000173 485	0,001299 605
ENSG0000010 8179	PPIF	peptidylprolyl isomerase F	-0,457124473	8,53E-11	2,79E-09

ENSG00000185608	MRPL40	mitochondrial ribosomal protein L40	-0,457206639	0,001494296	0,008387176
ENSG00000110955	ATP5B	ATP synthase%2C H+ transporting%2C mitochondrial F1 complex%2C beta polypeptide	-0,45869535	4,39E-19	5,83E-17
ENSG00000170855	TRIAP1	TP53 regulated inhibitor of apoptosis 1	-0,459008907	0,000506405	0,003309484
ENSG00000132313	MRPL35	mitochondrial ribosomal protein L35	-0,46012129	0,0000159	0,000161556
ENSG00000099800	TIMM13	translocase of inner mitochondrial membrane 13	-0,463395815	0,00000231	0,0000286
ENSG00000165688	PMPCA	peptidase%2C mitochondrial processing alpha subunit	-0,463833262	2,41E-08	0,000000477
ENSG00000265354	TIMM23	translocase of inner mitochondrial membrane 23	-0,467368776	0,00000109	0,0000145
ENSG00000109519	GRPEL1	GrpE like 1%2C mitochondrial	-0,471797731	0,00000122	0,0000161
ENSG00000117592	PRDX6	peroxiredoxin 6	-0,472765164	5,86E-11	2,01E-09
ENSG00000162377	COA7	cytochrome c oxidase assembly factor 7 (putative)	-0,473139077	0,000000104	0,00000177
ENSG00000143314	MRPL24	mitochondrial ribosomal protein L24	-0,474624513	0,00000807	0,0000876
ENSG00000010256	UQCRC1	ubiquinol-cytochrome c reductase core protein 1	-0,477792711	4,77E-13	2,52E-11
ENSG00000101365	IDH3B	isocitrate dehydrogenase 3 (NAD(+)) beta	-0,477939333	0,000013	0,000135858
ENSG00000156928	MALSU1	mitochondrial assembly of ribosomal large subunit 1	-0,478359555	0,000970515	0,00576961
ENSG00000188917	TRMT2B	tRNA methyltransferase 2 homolog B	-0,478708453	0,000301223	0,002110002
ENSG00000042286	AIFM2	apoptosis inducing factor%2C mitochondria associated 2	-0,47917239	0,001319243	0,007523341
ENSG00000050393	MCUR1	mitochondrial calcium uniporter regulator 1	-0,479552696	0,002184276	0,011527444
ENSG00000162972	MAIP1	matrix AAA peptidase interacting protein 1	-0,480718096	0,006393615	0,027985622
ENSG00000174032	SLC25A30	solute carrier family 25 member 30	-0,48372499	0,0000393	0,00036149
ENSG00000179091	CYC1	cytochrome c1	-0,48473705	1,2E-12	5,9E-11
ENSG00000150779	TIMM8B	translocase of inner mitochondrial membrane 8 homolog B	-0,485815838	0,000603852	0,003858565
ENSG00000117118	SDHB	succinate dehydrogenase complex iron sulfur subunit B	-0,486675511	0,00000515	0,0000585
ENSG00000027001	MIPEP	mitochondrial intermediate peptidase	-0,489807026	0,006084043	0,026817877
ENSG00000137124	ALDH1B1	aldehyde dehydrogenase 1 family member B1	-0,492126862	5,66E-08	0,00000103
ENSG00000166411	IDH3A	isocitrate dehydrogenase 3 (NAD(+)) alpha	-0,492954879	0,00000784	0,0000853

ENSG00000151552	QDPR	quinoid dihydropteridine reductase	-0,497275408	0,000167447	0,001265906
ENSG00000134375	TIMM17A	translocase of inner mitochondrial membrane 17A	-0,500170131	0,000000347	0,00000525
ENSG00000156502	SUPV3L1	Suv3 like RNA helicase	-0,500587454	0,0000111	0,000117121
ENSG00000154814	OXNAD1	oxidoreductase NAD binding domain containing 1	-0,50096684	0,000468239	0,00308302
ENSG00000137513	NARS2	asparaginyl-tRNA synthetase 2%2C mitochondrial	-0,502778679	0,00424053	0,020069706
ENSG00000110090	CPT1A	carnitine palmitoyltransferase 1A	-0,502896227	5,66E-13	2,96E-11
ENSG00000011376	LARS2	leucyl-tRNA synthetase 2%2C mitochondrial	-0,504684929	0,00000108	0,0000144
ENSG00000111666	CHPT1	choline phosphotransferase 1	-0,505365576	0,000288749	0,002035628
ENSG00000213339	QTRT1	queuine tRNA-ribosyltransferase catalytic subunit 1	-0,505517328	0,000462383	0,00305977
ENSG00000111639	MRPL51	mitochondrial ribosomal protein L51	-0,509346605	0,00011224	0,000897512
ENSG00000122140	MRPS2	mitochondrial ribosomal protein S2	-0,510930106	0,00000165	0,0000211
ENSG00000151729	SLC25A4	solute carrier family 25 member 4	-0,512777403	0,01166989	0,045376394
ENSG00000105953	OGDH	oxoglutarate dehydrogenase	-0,513649261	6,88E-10	0,000000019
ENSG00000122873	CISD1	CDGSH iron sulfur domain 1	-0,517257271	0,000710943	0,004415966
ENSG00000138035	PNPT1	polyribonucleotide nucleotidyltransferase 1	-0,526123928	6,43E-09	0,000000144
ENSG00000178952	TUFM	Tu translation elongation factor%2C mitochondrial	-0,52615019	7,11E-16	5,81E-14
ENSG00000008394	MGST1	microsomal glutathione S-transferase 1	-0,526922511	0,00000659	0,0000732
ENSG00000175756	AURKAIP1	aurora kinase A interacting protein 1	-0,527172233	0,000000701	0,00000989
ENSG00000125166	GOT2	glutamic-oxaloacetic transaminase 2	-0,527650332	7,42E-10	2,02E-08
ENSG00000116688	MFN2	mitofusin 2	-0,528164264	2,7E-10	8,13E-09
ENSG00000065057	NTHL1	nth like DNA glycosylase 1	-0,529043028	0,002140814	0,011348127
ENSG00000104907	TRMT1	tRNA methyltransferase 1	-0,529289421	0,00000143	0,0000184
ENSG00000164933	SLC25A32	solute carrier family 25 member 32	-0,529299617	0,00000009	0,00000155
ENSG00000182154	MRPL41	mitochondrial ribosomal protein L41	-0,530287111	0,00000063	0,00000904
ENSG00000103356	EARS2	glutamyl-tRNA synthetase 2%2C mitochondrial	-0,530584019	0,00000114	0,000015
ENSG00000105364	MRPL4	mitochondrial ribosomal protein L4	-0,53101132	0,000000355	0,00000537

ENSG00000183010	PYCR1	pyrroline-5-carboxylate reductase 1	-0,535635438	0,0000126	0,00013168
ENSG00000175606	TMEM70	transmembrane protein 70	-0,537080621	0,00000275	0,0000334
ENSG00000128654	MTX2	metaxin 2	-0,538081009	0,000310052	0,002160351
ENSG00000147684	NDUFB9	NADH:ubiquinone oxidoreductase subunit B9	-0,540650751	5,45E-10	1,55E-08
ENSG00000116221	MRPL37	mitochondrial ribosomal protein L37	-0,541714286	7,82E-17	7,3E-15
ENSG00000127884	ECHS1	enoyl-CoA hydratase%2C short chain 1	-0,544538823	1,62E-09	4,12E-08
ENSG00000186603	HPDL	4-hydroxyphenylpyruvate dioxygenase like	-0,549244588	0,001899081	0,010269391
ENSG00000247077	PGAM5	PGAM family member 5%2C mitochondrial serine/threonine protein phosphatase	-0,549417275	4,33E-12	1,83E-10
ENSG00000171421	MRPL36	mitochondrial ribosomal protein L36	-0,551070324	0,000156823	0,001199104
ENSG00000123545	NDUFAF4	NADH:ubiquinone oxidoreductase complex assembly factor 4	-0,551255559	0,000314401	0,002187184
ENSG00000147586	MRPS28	mitochondrial ribosomal protein S28	-0,555204756	0,007730902	0,032665853
ENSG00000004142	POLDIP2	DNA polymerase delta interacting protein 2	-0,55834936	4,25E-12	1,81E-10
ENSG00000127838	PNKD	paroxysmal nonkinesigenic dyskinesia	-0,558684493	0,002229621	0,011729126
ENSG00000185818	NAT8L	N-acetyltransferase 8 like	-0,559653168	7,34E-10	0,00000002
ENSG00000004779	NDUFAB1	NADH:ubiquinone oxidoreductase subunit AB1	-0,562264302	4,96E-09	0,000000114
ENSG00000125445	MRPS7	mitochondrial ribosomal protein S7	-0,564070906	0,000000114	0,00000192
ENSG00000059573	ALDH18A1	aldehyde dehydrogenase 18 family member A1	-0,564558142	4,26E-12	1,81E-10
ENSG00000100116	GCAT	glycine C-acetyltransferase	-0,566467697	0,000196944	0,001451387
ENSG00000130299	GTPBP3	GTP binding protein 3%2C mitochondrial	-0,566608713	0,000225067	0,001632141
ENSG00000075336	TIMM21	translocase of inner mitochondrial membrane 21	-0,566772002	0,00000774	0,0000845
ENSG00000118246	FASTKD2	FAST kinase domains 2	-0,569509593	0,000000669	0,0000095
ENSG00000172115	CYCS	cytochrome c%2C somatic	-0,569743164	4,38E-13	2,33E-11
ENSG00000099821	POLRMT	RNA polymerase mitochondrial	-0,570279443	1,61E-08	0,000000331
ENSG00000183048	SLC25A10	solute carrier family 25 member 10	-0,572863643	0,0000399	0,000366447
ENSG00000150768	DLAT	dihydrolipoamide S-acetyltransferase	-0,572990534	1,26E-09	3,28E-08

ENSG00000156026	MCU	mitochondrial calcium uniporter	-0,573797077	0,00000004	0,000000755
ENSG00000114023	FAM162A	family with sequence similarity 162 member A	-0,575439277	0,0000929	0,0007609
ENSG00000196449	YRDC	yrdC N6-threonylcarbamoyltransferase domain containing	-0,57581618	0,005756981	0,025661633
ENSG00000123131	PRDX4	peroxiredoxin 4	-0,576910799	0,000000226	0,00000356
ENSG00000134326	CMPK2	cytidine/uridine monophosphate kinase 2	-0,5769785	0,0000738	0,000621188
ENSG00000137824	RMDN3	regulator of microtubule dynamics 3	-0,581463576	0,000000626	0,000009
ENSG00000143436	MRPL9	mitochondrial ribosomal protein L9	-0,58443107	4,33E-08	0,000000811
ENSG00000151726	ACSL1	acyl-CoA synthetase long chain family member 1	-0,586873188	3,6E-11	1,3E-09
ENSG00000114686	MRPL3	mitochondrial ribosomal protein L3	-0,587107798	3,32E-14	2,11E-12
ENSG00000177192	PUS1	pseudouridylate synthase 1	-0,587848973	0,000000325	0,00000494
ENSG00000072506	HSD17B10	hydroxysteroid 17-beta dehydrogenase 10	-0,593278251	2,59E-09	6,39E-08
ENSG00000197785	ATAD3A	ATPase family%2C AAA domain containing 3A	-0,594315717	0,00000112	0,0000148
ENSG00000108561	C1QBP	complement C1q binding protein	-0,594858004	5,39E-14	3,3E-12
ENSG00000158042	MRPL17	mitochondrial ribosomal protein L17	-0,595859867	0,000000127	0,00000211
ENSG00000162910	MRPL55	mitochondrial ribosomal protein L55	-0,596007446	0,000123718	0,000973329
ENSG00000159199	ATP5G1	ATP synthase%2C H+ transporting%2C mitochondrial Fo complex subunit C1 (subunit 9)	-0,610218309	2,69E-08	0,000000527
ENSG00000172590	MRPL52	mitochondrial ribosomal protein L52	-0,618095744	9,32E-09	0,000000201
ENSG00000258429	PDF	peptide deformylase%2C mitochondrial	-0,620272713	0,005825165	0,02591106
ENSG00000105607	GCDH	glutaryl-CoA dehydrogenase	-0,620430196	0,000000296	0,00000453
ENSG00000151806	GUF1	GUF1 homolog%2C GTPase	-0,621211776	1,18E-10	3,78E-09
ENSG00000115541	HSPE1	heat shock protein family E (Hsp10) member 1	-0,625339201	0,012533799	0,048124231
ENSG00000035141	FAM136A	family with sequence similarity 136 member A	-0,625524779	1,33E-08	0,000000277
ENSG00000150756	FAM173B	family with sequence similarity 173 member B	-0,62848013	0,00000277	0,0000336
ENSG00000160124	CCDC58	coiled-coil domain containing 58	-0,631715954	0,000230997	0,001669622
ENSG00000103160	HSDL1	hydroxysteroid dehydrogenase like 1	-0,636352192	0,001302099	0,007446122

ENSG0000001 3306	SLC25A39	solute carrier family 25 member 39	-0,636542703	1,21E-17	1,27E-15
ENSG0000011 6771	AGMAT	agmatinase	-0,638215045	0,000663 191	0,004170 531
ENSG0000012 8050	PAICS	phosphoribosylaminoimidazole carboxylase and phosphoribosylaminoimidazolesuccino carboxamide synthase	-0,638366898	1,37E-26	4,4E-24
ENSG0000013 9180	NDUFA9	NADH:ubiquinone oxidoreductase subunit A9	-0,640332484	0,003147 214	0,015628 405
ENSG0000024 2485	MRPL20	mitochondrial ribosomal protein L20	-0,6416387	7,92E-08	0,000001 4
ENSG0000014 8459	PDSS1	decaprenyl diphosphate synthase subunit 1	-0,653153622	0,000007 95	0,000086 4
ENSG0000015 6398	SFXN2	sideroflexin 2	-0,653788004	1,53E-08	0,000000 316
ENSG0000015 2620	NADK2	NAD kinase 2%2C mitochondrial	-0,657966016	9,14E-10	2,43E-08
ENSG0000016 9288	MRPL1	mitochondrial ribosomal protein L1	-0,661440876	0,000000 115	0,000001 92
ENSG0000015 6170	NDUFAF6	NADH:ubiquinone oxidoreductase complex assembly factor 6	-0,665548061	0,000147 048	0,001131 84
ENSG0000016 5283	STOML2	stomatin like 2	-0,666487565	2,26E-14	1,49E-12
ENSG0000017 9271	GADD45 GIP1	GADD45G interacting protein 1	-0,667495237	8,82E-12	3,57E-10
ENSG0000011 6096	SPR	sepiapterin reductase	-0,669580829	0,000008 19	0,000088 7
ENSG0000010 5197	TIMM50	translocase of inner mitochondrial membrane 50	-0,673420824	3,09E-14	2E-12
ENSG0000015 9685	CHCHD6	coiled-coil-helix-coiled-coil-helix domain containing 6	-0,677155587	0,005093 468	0,023334 572
ENSG0000018 5875	THNSL1	threonine synthase like 1	-0,678819718	0,004742 202	0,021970 034
ENSG0000013 2541	RIDA	reactive intermediate imine deaminase A homolog	-0,693752285	0,000001 51	0,000019 4
ENSG0000016 8268	NT5DC2	5'-nucleotidase domain containing 2	-0,701637594	1,85E-12	8,77E-11
ENSG0000012 6602	TRAP1	TNF receptor associated protein 1	-0,706769771	1,92E-21	3,16E-19
ENSG0000010 0216	TOMM22	translocase of outer mitochondrial membrane 22	-0,711488887	1,11E-18	1,38E-16
ENSG0000011 2651	MRPL2	mitochondrial ribosomal protein L2	-0,712395306	4,49E-08	0,000000 834
ENSG0000016 4182	NDUFAF2	NADH:ubiquinone oxidoreductase complex assembly factor 2	-0,722051843	0,000841 683	0,005126 435
ENSG0000018 6205	MARC1	mitochondrial amidoxime reducing component 1	-0,72894668	0,000013 3	0,000138 114
ENSG0000016 3170	BOLA3	bolA family member 3	-0,732673109	5,18E-08	0,000000 95
ENSG0000011 3013	HSPA9	heat shock protein family A (Hsp70) member 9	-0,735464237	3,35E-31	2E-28

ENSG00000224877	NDUFAF8	NADH:ubiquinone oxidoreductase complex assembly factor 8	-0,740684071	2,64E-08	0,000000518
ENSG00000262814	MRPL12	mitochondrial ribosomal protein L12	-0,743029215	0,00000105	0,000014
ENSG00000169230	PRELID1	PRELI domain containing 1	-0,752691223	7,3E-11	2,46E-09
ENSG00000137547	MRPL15	mitochondrial ribosomal protein L15	-0,758061063	2,67E-15	2E-13
ENSG00000119705	SLIRP	SRA stem-loop interacting RNA binding protein	-0,761742855	6,69E-11	2,28E-09
ENSG00000137038	DMAC1	distal membrane arm assembly complex 1	-0,77282754	7,45E-09	0,000000165
ENSG00000025772	TOMM34	translocase of outer mitochondrial membrane 34	-0,777506221	8,4E-14	5E-12
ENSG00000076043	REXO2	RNA exonuclease 2	-0,778490012	0,00000724	0,0000798
ENSG00000104980	TIMM44	translocase of inner mitochondrial membrane 44	-0,791115841	1,12E-13	6,43E-12
ENSG00000197345	MRPL21	mitochondrial ribosomal protein L21	-0,801765392	8,67E-11	2,83E-09
ENSG00000130204	TOMM40	translocase of outer mitochondrial membrane 40	-0,817442391	1,12E-25	3,13E-23
ENSG00000135002	RFK	riboflavin kinase	-0,82623642	3,99E-12	1,71E-10
ENSG00000167085	PHB	prohibitin	-0,831675749	5,96E-14	3,62E-12
ENSG00000102743	SLC25A15	solute carrier family 25 member 15	-0,842579757	4,52E-11	1,58E-09
ENSG00000134809	TIMM10	translocase of inner mitochondrial membrane 10	-0,852269177	1,7E-10	5,32E-09
ENSG00000144381	HSPD1	heat shock protein family D (Hsp60) member 1	-0,865518031	7,94E-13	4,07E-11
ENSG00000116791	CRYZ	crystallin zeta	-0,869340765	0,00047307	0,003113272
ENSG00000074582	BCS1L	BCS1 homolog%2C ubiquinol-cytochrome c reductase complex chaperone	-0,869651472	4,24E-09	9,92E-08
ENSG00000120254	MTHFD1L	methylenetetrahydrofolate dehydrogenase (NADP+ dependent) 1 like	-0,913096583	1,41E-26	4,41E-24
ENSG00000123213	NLN	neurolysin	-1,110228923	9,86E-34	8,11E-31
